# Revisiting the Rist diagram for predicting operating conditions in blast furnaces with multiple injections

**DOI:** 10.12688/openreseurope.14275.1

**Published:** 2021-11-29

**Authors:** Manuel Bailera, Takao Nakagaki, Ryoma Kataoka

**Affiliations:** 1 Graduate School of Creative Science and Engineering, Waseda University, Tokyo, 1698555, Japan; 2 Escuela de Ingeniería y Arquitectura, University of Zaragoza, Zaragoza, 50018, Spain; 3 Department of Modern Mechanical Engineering, Waseda University, Tokyo, 1698555, Japan; 4 Exergy Engineering Research, Nakagaki Lab, Waseda University, Tokyo, 1698555, Japan

**Keywords:** Blast furnace, Rist diagram, ironmaking, operating diagram, Carbon capture, Power to Gas, Oxyfuel combustion, CO2

## Abstract

**Background:** The Rist diagram is useful for predicting changes in blast furnaces when the operating conditions are modified. In this paper, we revisit this methodology to provide a general model with additions and corrections. The reason for this is to study a new concept proposal that combines oxygen blast furnaces with Power to Gas technology. The latter produces synthetic methane by using renewable electricity and CO _2_ to partly replace the fossil input in the blast furnace. Carbon is thus continuously recycled in a closed loop and geological storage is avoided.

**Methods:** The new model is validated with three data sets corresponding to (1) an air-blown blast furnace without auxiliary injections, (2) an air-blown blast furnace with pulverized coal injection and (3) an oxygen blast furnace with top gas recycling and pulverized coal injection. The error is below 8% in all cases.

**Results:** Assuming a 280 t _HM_/h oxygen blast furnace that produces 1154 kg _CO2_/t _HM_, we can reduce the CO _2_ emissions between 6.1% and 7.4% by coupling a 150 MW Power to Gas plant. This produces 21.8 kg/t _HM_ of synthetic methane that replaces 22.8 kg/t _HM_ of coke or 30.2 kg/t _HM_ of coal. The gross energy penalization of the CO _2_ avoidance is 27.1 MJ/kg _CO2_ when coke is replaced and 22.4 MJ/kg _CO2_ when coal is replaced. Considering the energy content of the saved fossil fuel, and the electricity no longer consumed in the air separation unit thanks to the O _2_ coming from the electrolyzer, the net energy penalizations are 23.1 MJ/kg _CO2_ and 17.9 MJ/kg _CO2_, respectively.

**Discussion:** The proposed integration has energy penalizations greater than conventional amine carbon capture (typically 3.7 – 4.8 MJ/kg _CO2_), but in return it could reduce the economic costs thanks to diminishing the coke/coal consumption, reducing the electricity consumption in the air separation unit, and eliminating the requirement of geological storage.

## Plain language summary

The steel industry is one of the most CO _2_ emitting industries worldwide. In this article we study the possibility of recycling the CO _2_ that is emitted in order to produce natural gas. Thus, the CO _2_ emissions are converted to a useful fuel instead of released to the atmosphere. For this process, renewable electricity is used, so the natural gas produced can be considered environmentally friendly. With this natural gas we want to replace the fossil fuel that is conventionally used in the steel industry. To make this study we have used a mathematical model that was developed by a researcher named Rist between 1963 and 1967. We made some corrections and additions to this mathematical model to make it more accurate. The results of this study show that the CO _2_ emissions can be reduced between 6.1% and 7.4% by using commercially available technology.

## 1. Introduction

The potential contribution of carbon capture and utilization to the global warming mitigation challenge has shown to be very limited when compared with geological storage or electrification ^1, 2^. If we talk in particular about e-fuels ( *e.g.*, hydrogen from renewables and synthetic methane), the electricity-to-useful-energy efficiencies range from roughly 10% to 35%, meaning that energy requirements are 2–14-times higher than for direct electrification ^2^. However, some of the most energy- and carbon-intensive sectors worldwide face limitations when applying electrification. In some cases, this is because the requirement of high-temperature heat above 400°C ( *e.g.*, glass, cement) and others because the nature of the process itself ( *e.g.*, ironmaking, long-distance aviation and shipping) ^2^. Renewable hydrogen and synthetic fuels can overcome this barriers, delivering the same service at lower costs than the other CO _2_ abatement alternatives, so they should be targeted on these industries from an economic and carbon-neutrality perspective ^1, 2^. Furthermore, given the substantial size of the mentioned sectors, the application of e-fuels within them should be prioritized ^2^.

Within this framework, several authors have studied the application of power to gas (PtG) to ironmaking processes based on the reduction of iron ores with coke in a blast furnace (BF). The PtG concept includes all those processes that converts renewable electricity into gaseous fuel by using an electrolysis stage (among other steps) ^3^. In this case, the renewable fuel is used for the replacement of coke or coal in the blast furnace. According to the literature ^4^, the integrations involving power to syngas may lead to CO _2_ emission reductions between 11% and 22%, with respect to conventional blast furnaces. The required electricity consumption of the overall system, per kilogram of CO _2_ recycled, lies in the range 4.8–10.8 MJ/kg _CO2_. Moreover, the thermal energy necessities vary from 1 to 2.5 MJ/kg _CO2_, increasing to 7.8 MJ/kg _CO2_ if carbon capture is used. The electrolysis power capacity required in this integrations range between 100 MW and 900 MW.

Regarding the integration of ironmaking with power to methane, the available studies in literature are very scarce. In these studies ^5, 6^, the CO _2_ emissions’ reduction compared with conventional ironmaking is in the range 13%–19%. Even for these moderate reductions, water electrolysis power capacities of about 880 MW would be required. Additionally, Bailera *et al.* ^4^ proposed a novel concept that combines power to methane with oxygen blast furnaces (OBF). In the OBF, pure oxygen is used for combustion instead of air, thus obtaining a top gas with very little nitrogen. In this type of blast furnace, it is usual to separate the CO _2_ from the top gas and to recycle the H _2_ and CO content again to the blast furnace to act as reducing agents and as a heat sink (because N _2_ is no longer present) ^7^. Since water electrolysis of the PtG process by-produces O _2_, it allows diminishing the electricity consumption of the air separation unit that feeds the OBF. A first approach to this OBF–PtG system was studied by Perpiñán *et al.* ^8^ by using overall energy and mass balances. Assuming 430 MW electrolysis power capacity, they found a CO _2_ emission reduction of 8% and specific electricity consumptions of 34 MJ/kg _CO2_.

In order to deep in the concept of OBF–PtG integration, a more detailed analysis of the behavior of the blast furnace is required. To do so, the Rist diagram (also known as the operating diagram) is a convenient methodology for predicting changes in blast furnaces when the operating conditions are modified. This methodology is based on the graphical representation of carbon, oxygen, and hydrogen balances through an operation line, restricted by the energy balance, which depicts the participation of these elements in the formation of the reducing gas and its later utilization inside the furnace ^9^. The original model is thoroughly explained in a series of papers that progressively deeps into the topic ^9– 13^. However, some of the most important parts were not written in English, and a paper summarizing the general model is not available. As a result, relevant aspects of his work are sometimes not widely known. Such is the case that some authors claim to modify the Rist diagram to include the H _2_ contribution ^14, 15^, when in fact this was already taken into account by Rist. For these reasons, we decided to revisit his original work, during which we made a number of additions and corrections.

Thus, the first major novelty of this paper is presenting a general operating diagram methodology that considers multiple injectants treated separately, with all calculations given as a function of the temperature of the thermal reserve zone that exists inside the furnace. Besides, the new model calculates the sensible heat of the hot metal and slag as a function of their composition, and the heat of carburization as a function of the austenite and cementite content in iron. Furthermore, it is added a supplementary model to compute the heat of decomposition of coal, an additional energy balance in the upper zone of the blast furnace to compute the final composition of the blast furnace gas (BFG), as well as other energy balance for the calculation of the flame temperature. Regarding corrections with respect to the Rist’s original model, the heat associated with the direct reduction of FeO now accounts for the moisture of the hot blast, the heat associated with the lack of chemical ideality now includes the influence of the hydrogen coming from auxiliary fuels and of the moisture of the hot blast, and lastly, the sensible heat of hot metal and slag are now correctly computed and accounted.

The second major novelty of the paper is analyzing for the first time the OBF–PtG integration under the operating diagram methodology and, therefore, by using consistent operation data sets. Besides, the operating lines of these blast furnaces are obtained, which cannot be found elsewhere in literature. The third major novelty is to provide full operation data sets for different blast furnace, with detailed composition of all streams and their most relevant operating parameters ( *e.g*., temperature of the thermal reserve zone, heat evacuated by the staves, and the temperature of the flame). The availability of this information in literature is very scarce, especially for OBFs.

The paper is divided in the following sections. First, a brief description of a blast furnace is presented to summarize the processes that will be taken into account during the elaboration of the operating diagram and in the calculation of the operating line ( Section 2). Then, the construction of the operating diagram ( Section 3) and the calculation methodology of the operating line ( Section 4) are thoroughly described, highlighting the new contributions with respect to the original work of Rist. The model is validated with different data sets elaborated from literature data ( Section 5), and then used to obtain new operating lines of oxygen blast furnaces with synthetic natural gas injection ( Section 6). The paper also includes an exhaustive section of appendixes to make the proposed methodology and the obtained results fully reproducible by the reader. Moreover, the same notation as Rist has been used in order to make easier the comparison between both methodologies.

## 2. Blast furnace

The largest blast furnaces at present can produce 10–13 kt of hot metal a day. They are about 34 m in inner height (distance from the raw material entrance to the hot metal exit) and 16 m in diameter, with an internal volume in the range 5000–5500 m ^3^ ^16, 17^. The blast furnace has a vertical cylindrical structure, externally covered with a shell of steel and internally with refractories. Between the shell and the refractories, the structure is cooled by staves ^16^. Staves are cooling gadgets having one or more inside channels through which water flows. The heat removed by cooling may be about 400–1800 MJ/thm ^10, 17– 20^.

**Figure 1.**
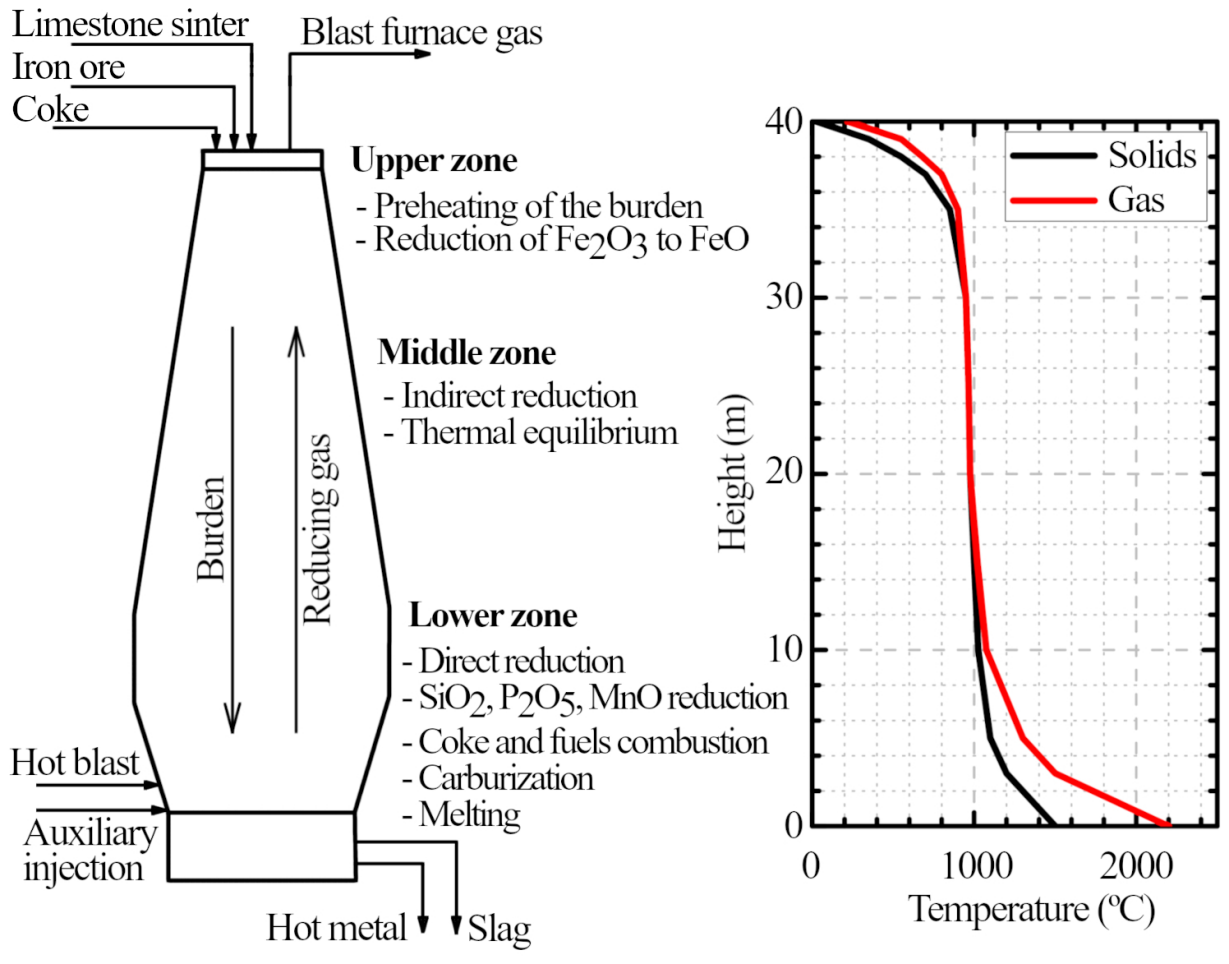
Schematic diagram of a blast furnace and its typical temperature profile.

Iron ore and coke, which are introduced at the top, take 5–7 hours to descend to the bottom by gravity ^21^. To reduce this burden, a reducing gas (mainly CO, but also H _2_) ascends throughout the furnace in 5–10 seconds and reduces iron ores after going through numerous chemical reactions ( Figure 1). The gas is produced at the lower part of the furnace by burning the coke with O _2_-enriched pressurized air injected through the tuyeres (coke is the only charged material which descends to the tuyere level in the solid state). The gases move upward due to the pressure of this hot blast and exit the furnace at the top at 2.0–2.5 bar. Auxiliary fuels, such as pulverized coal or natural gas, can also be injected through the tuyeres to diminish the amount of coke introduced with the burden. At the bottom, the molten metal is collected ^16^.

### 2.1 Chemical reactions

Inside the blast furnace, the process is as follows ( Figure 1). In the upper part (4–6 m from the top), hematite is reduced to magnetite through irreversible reactions ( Eq.(1) and Eq.(2), exothermic), and then to wüstite ( Eq.(3) and Eq.(4), endothermic) ^16^. To ensure the reduction, the ratios of CO/CO _2_ and H _2_/H _2_O have to exceed their stoichiometric value. Otherwise, the reactions would not achieve equilibrium because of the short residence time of the gases in the furnace.


$$\;3{\text{Fe}}_{2}{\text{O}}_{3}+\text{CO}\to 2{\text{Fe}}_{3}{\text{O}}_{4}+{\text{CO}}_{2}\;\text{ΔH}=-52.85\;\text{kJ}/\text{mol}\;(1)$$

$$\;{\text{3Fe}}_{2}{\text{O}}_{3}+{\text{H}}_{\text{2}}\to 2{\text{Fe}}_{\text{3}}{\text{O}}_{\text{4}}+{\text{H}}_{\text{2}}\text{O}\;\text{ΔH}=-\;4.86\;\text{kJ}/\text{mol}\;(2)$$

$$\;{\text{Fe}}_{3}{\text{O}}_{4}+\text{CO}\to 3\text{FeO}+{\text{CO}}_{2}\;\text{ΔH}=+36.46\;\text{kJ}/\text{mol}\;(3)$$

$$\;{\text{Fe}}_{3}{\text{O}}_{4}+{\text{H}}_{\text{2}}\to 3\text{FeO}+{\text{H}}_{\text{2}}\text{O}\;\text{ΔH}=+84.45\;\text{kJ}/\text{mol}\;(4)$$

The middle zone of the blast furnace extends about 25 m downward from the end of the upper zone. It takes 2.5–3 hours for the burden to traverse the zone, during which the wüstite is partially reduced following Eq.(5) and Eq.(6) (indirect reduction) ^16^. This process is exothermic for CO and endothermic for H _2_. In addition, the water–gas shift reaction reaches equilibrium in this zone, Eq.(7) ^20^.


$$\;\text{FeO}+\text{CO}\to \text{Fe}+{\text{CO}}_{\text{2}}\;\text{ΔH}=-17.13\;\text{kJ}/\text{mol}\;(5)$$

$$\;\text{FeO}+{\text{H}}_{\text{2}}\to \text{Fe}+{\text{H}}_{\text{2}}\text{O}\;\text{ΔH}=+30.86\;\text{kJ}/\text{mol}\;(6)$$

$$\;\text{CO}+{\text{H}}_{\text{2}}\text{O}↔{\text{CO}}_{\text{2}}+{\text{H}}_{\text{2}}\;\text{ΔH}=-40.45\;\text{kJ}/\text{mol}\;(7)$$

In the lower zone (3–5 m above the tuyeres to the bottom), the rest of the wüstite is reduced by coke carbon through the direct reduction process ( Eq.(8), endothermic) ^16^. Actually, the interaction between iron and coke is limited, but at temperatures above 1000°C the CO _2_ and H _2_O reacts with coke and forms CO and H _2_ ( Eq.(9) and Eq.(10)), which subsequently reduce the iron oxide by Eq.(5) and Eq.(6). This way, coke is consumed ^16^.


FeO+C→Fe+COΔH=+155.34kJ/mol(8)

$$\;{\text{CO}}_{\text{2}}+\text{C}\to 2\text{CO}\;\text{ΔH}=+172.47\;\text{kJ}/\text{mol}\;(9)$$

$$\;{\text{H}}_{\text{2}}\text{O}+\text{C}\to {\text{H}}_{\text{2}}+\text{CO}\;\text{ΔH}=+124.48\;\text{kJ}/\text{mol}\;(10)$$

Apart from the direct reduction, other relevant processes occur in the lower zone. The burden contains various impurities that will either dissolve in iron or will form part of the slag. For example, the Al _2_O _3_, CaO and MgO oxides are not reduced under the blast furnace conditions and therefore they transfer fully into the slag. In the case of SiO _2_, MnO and P _2_O _5_, they are partially reduced and dissolved in the hot metal. It can be considered that these impurities are directly reduced by solid carbon following Eq.(11), Eq.(12) and Eq.(13). The final silicon, manganese and phosphorous contents in the hot metal are much less than the equilibrium values.


$$\;{\text{SiO}}_{\text{2}}+2\text{C}+3\text{Fe}\to {\text{Fe}}_{\text{3}}\text{Si}+2\text{CO}\;\text{ΔH}=+622.5\;\text{kJ}/\text{mol}\;(11)$$

MnO+C→Mn+COΔH=+286.92kJ/mol(12)

$$\;{\text{P}}_{\text{2}}{\text{O}}_{5}\cdot 3\text{CaO}+5\text{C}+6\text{Fe}\to 2{\text{Fe}}_{\text{3}}\text{P}+3\text{CaO}+5\text{CO}\;\text{ΔH}=+1184\;\text{kJ}/\text{mol}\;(13)$$

Another component that will end up dissolved in the hot metal is carbon. As in the previous case, carbon never reaches saturation in pig iron ^20^. At tapping temperatures (1350–1450°C) the carbon content may vary from 2.5 to 4.5% ^16^. As simplification, it is assumed that the dissolved carbon forms, austenite and cementite, in the hot metal, according to Eq.(14) and Eq.(15), respectively ^11^.


C(coke)→C(austenite)ΔH=+34.7kJ/mol(14)

$$\;3\text{Fe}+\text{C}(\text{coke})\to {\text{Fe}}_{\text{3}}\text{C}\;\text{ΔH}=+6.69\;\text{kJ}/\text{mol}\;(15)$$

Also, in the lower zone, in front of the tuyeres ( *i.e*., the raceway), coke burns with the oxygen of the hot blast, thus providing the process with heat and CO-reducing gas. The total reaction of coke in the raceway can be considered as an incomplete combustion due to the shortage of oxygen ( Eq.(16)) ^16^. Actually, in the inner part of the flame, complete combustion also occurs but the CO _2_ ends up dissociating by Eq.(9).


$$\;\text{C}+\frac{1}{2}{\text{O}}_{2}\to \text{CO}\;\text{ΔH}=-113.68\;\text{kJ}/\text{mol}\;(16)$$

In case of injecting auxiliary fuels to diminish coke consumption, incomplete combustion is assumed to follow Eq.(17), where Z denotes the ashes in the case of pulverized coal. No water is present since it rapidly dissociates by Eq.(10) as it occurred for CO _2_^16^.


$$\;{\text{CH}}_{2\text{a}}{\text{O}}_{2\text{b}}{\text{N}}_{2\text{c}}{\text{S}}_{2\text{d}}{\text{Z}}_{\text{z}}+\frac{1}{2}{\text{O}}_{2}\to \text{CO}+{\text{aH}}_{2}+{\text{bO}}_{2}+{\text{cN}}_{2}+{\text{dS}}_{2}+\text{zZ}\;(17)$$

When the injected fuel contains sulfur ( *e.g.*, pulverized coal), this will end forming part of the slag. As simplification, it can be considered that sulfur dissolves into the hot metal by Eq.(18), and then transfers to the slag by Eq.(19) ^20^.


$$\;\text{Fe}+0.5{\text{S}}_{2}\to \text{FeS}\;(18)$$

FeS+CaO+C→Fe+CaS+CO(19)

Per mole of S that ends in the slag, 1 mole of CO is added to the reducing gas.

### 2.2 Temperature profile

The blast furnace process can be divided in three different temperatures zones ( Figure 1). At the lower part, the flame temperature is normally between 2000 and 2300°C (defined as the temperature reached by the raceway gas when all C and H _2_O have been converted to CO and H _2_) ^22^. This raceway gas provides heat for the direct reduction process and for the melting of the hot metal and slag ^20^. The hot metal exits at 1350–1450°C and the slag at 1500–1550°C ^16^. The gas, which has been cooled to about 1000°C, ascends to the middle zone.

The middle zone is a region of thermal equilibrium. In practice, a non-zero temperature difference remains between gas and solids, but it passes through a minimum value in a region of slow heat exchange. The temperature is kept almost constant around 800–1000°C ^10^.

Lastly, in the upper zone, the gas and the burden exchanges heat rapidly. The gas is cooled down from 800–900°C to 100–200°C as it leaves the furnace top, and the burden is heated from ambient temperature to 800°C while descending ^16, 20^.

The temperature profile and the reduction zones (pre-reduction, indirect reduction, and direct reduction) more or less coincide, so these three zones can be used to study the blast furnace.

## 3. Generalized Rist diagram with multiple injectants

The Rist diagram is named so in reference to its author, who elaborated a model for predicting changes in blast furnaces when the operating conditions are modified ^9– 13^. The model is based on the graphical representation of carbon, oxygen, and hydrogen balances through an operation line that depicts the participation of these elements in the formation and utilization of the reducing gas ^9^. Additionally, the diagram includes an equilibrium line to delimit the maximum oxidation state of the gas according to the Chaudron diagrams for the Fe-O-H and Fe-O-C systems ^23^.

The construction of the Rist diagram is introduced here with additions and corrections with respect to the original work of Rist. The model methodology is now described for the general case of multiple injectants treated separately (instead of for an overall single injection). This is especially important because it will allow to properly calculate the heat of decomposition of each auxiliary fuel, as well as to specify different inlet temperatures for each injectant. Additionally, a detailed description on how to find the equilibrium line for the diagram is included.

### 3.1 Formation of the reducing gas (Rist diagram in the range 0<X<1)

The mass balance of the formation of 1 mol of reducing gas mixture ( *i.e.*, the gas exiting the lower zone) can be written according to Eq.(20) ^9^.


$$\;x_{v}+2x_{e}+\sum (a_{j}+2b_{j})x_{j}+x_{Si}+x_{Mn}+x_{P}+x_{S}+x_{k}+x_{d}=1\;\text{mol}\;\text{of}\;\text{reducing}\;\text{gas}\;(20)$$

In this equation, each addend denotes the number of moles of CO and/or H _2_ (per mole of the total reducing gas mixture) that are either introduced in the blast furnace or produced through a reaction. In other words, these addends are the individual contributions (in mole fractions) to the formation of the reducing gas mixture. They are given according to the sources of hydrogen (H _2_), oxygen (0.5 mole of O _2_ will give 1 mole of CO) or water (1 mole of H _2_O will give 1 mole of CO and 1 mole of H _2_).

The term *x _v_* denotes the CO produced when the O _2_ of the hot blast react with C through Eq.(16) or Eq.(17). The term 2 *x _e_* accounts for the H _2_ and CO produced by the moisture of the hot blast when dissociated through Eq.(10). The term *a _j_* *x _j_* denotes the H _2_ from the incomplete combustion of an auxiliary injection j ( Eq.(17)), while 2 *b _j_* *x _j_* is the CO produced when the oxygen of that auxiliary injection react with C according to Eq.(16). The terms *x _Si_*, *x _Mn_* and *x _p_* stand for the CO produced when reducing the impurities SiO _2_, MnO and P _2_O _5_ ( Eq.(11), Eq.(12) and Eq.(13)). The addend *x _S_* represents the CO released when transferring the dissolved sulfur in the iron to the slag ( Eq.(19)). The term *x _k_* is the H _2_ directly coming from the hydrogen content of the coke. Lastly, *x _d_* denotes the CO released during the direct reduction of wüstite ( Eq.(8)) ^10^.

**Figure 2.**
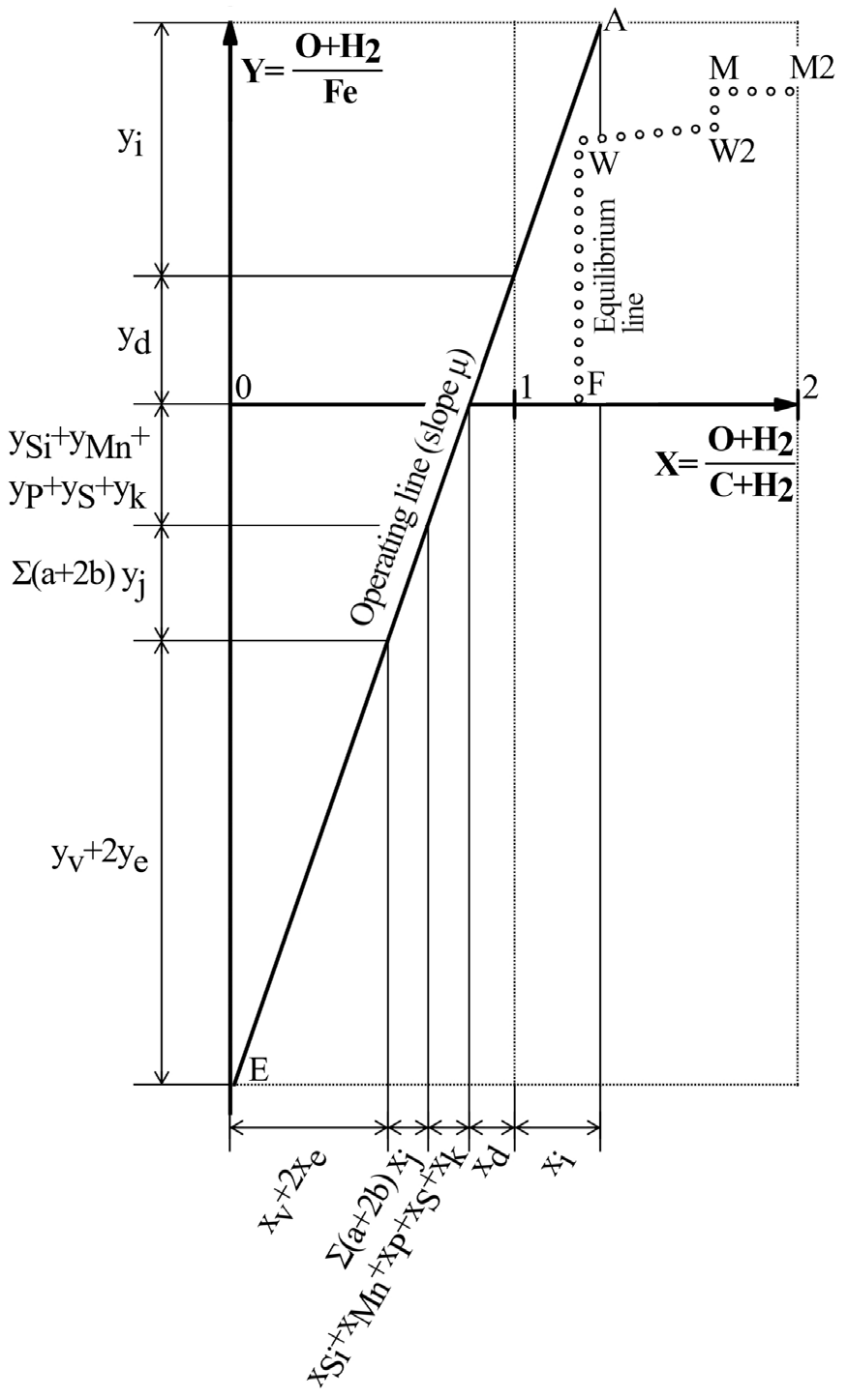
Rist diagram.

Each of the addends of Eq.(20) can be depicted as a segment on the abscissa axis, whose total sum covers the interval 0<X<1 ( Figure 2). For convenience, the notation of Eq.(21) is used for the units of the abscissas in the diagram, where the numerator is the number of moles of reducing gas related to a particular reaction or injection according to the sources of hydrogen (H _2_) and oxygen (0.5 mole of O _2_ giving 1 mole of CO), and the denominator is the total number of moles of reducing gas according to the sources of hydrogen (H _2_) and carbon (1 mole of C giving 1 mole of CO) ^12^.


$$\;\text{X}=\frac{\text{O}+{\text{H}}_{2}}{\text{C}+{\text{H}}_{2}}\;(21)$$

Alternatively, the mass balance for the production of the reducing gas mixture can also be written with reference to the production of 1 mole of Fe in the blast furnace. Under this reference, the mass balance follows Eq.(22).


$$y_{v}+2y_{e}+\sum (a_{j}+2b_{j})y_{j}+y_{Si}+y_{Mn}+y_{P}+y_{S}+y_{k}+y_{d}=\text{μ}\;\text{moles}\;\text{of}\;\text{reducing}\;{\text{gas/mol}}_{\text{Fe}}\;(22)$$

Here, the addends denote the moles of reducing gas (CO and H _2_) that are introduced in the blast furnace or produced through a reaction, per unit of Fe obtained (the meaning of each addend is identical to Eq.(20)) ^10^. The sum of these segments represents the total moles of reducing gas per unit of Fe (denoted by μ) ^9^. Each of the addends of Eq.(22) can be represented as a segment on the ordinate axis ( Figure 2). In this case, the origin on the Y axis of the operating diagram is arbitrary. For convenience, it is chosen so that the oxygen originally combined to iron appears on the positive side ( *i.e*., *y _d_*), whereas other sources of oxygen and hydrogen appear on the negative side (this ordinate is denoted by Y _E_ for convenience, Eq.(23)) ^9^.


$$\;{\text{Y}}_{\text{E}}=-\left(y_{v}+2y_{e}+\sum (a_{j}+2b_{j})y_{j}+y_{Si}+y_{Mn}+y_{P}+y_{S}+y_{k}\right)\;(23)$$

The units of the ordinate axis are set according to Eq.(24), given as a function of the sources of hydrogen (H _2_) and oxygen (0.5 mole of O _2_ giving 1 mole of CO) for the production of the reducing gas.


$$\;\text{Y}=\frac{\text{O}+{\text{H}}_{2}}{\text{Fe}}\;(24)$$

The corresponding terms of Eq.(20) and Eq.(22) form sets of proportional numbers and, as such, they can be read on two rectangular axes as the projections of the same straight line. The Figure 2 shows the straight line thus obtained, called the “operating line” ^9^, whose slope has the units of Eq.(25).


$$\;\text{μ}=\frac{\text{C}+{\text{H}}_{2}}{\text{Fe}}\;(25)$$

This corresponds to the total number of moles of reducing gas, per unit of Fe, according to the sources of hydrogen and carbon.

### 3.2 Utilization of the reducing gas (Rist diagram in the range 1<X<2)

Following the same principle, it is simple to depict the utilization of the reducing gas mixture ( Eq.(1) – Eq.(6)) by means of a segment representing the oxygen removed from the iron oxides. The number of oxygen moles transferred from the iron oxides to the reducing gas is denoted by *x _i_* when referred to 1 mole of reducing gas mixture, and by *y _i_* when referred to 1 mole of Fe (same convention than before). Thus, the ratio between both variables ( *i.e*., *y _i_*/ *x _i_*) is the number of moles of reducing gas mixture per mole of Fe produced. Since the reduction of iron oxides does not change the total number of moles of the reducing gas, the ratio *y _i_*/ *x _i_* is constant and equal to μ. In other words, in all equations from Eq.(1) to Eq.(6) the gas gets oxidized without increasing or decreasing the number of moles in the gas (1 mole of CO gives 1 mole of CO _2_, and 1 mole of H _2_ gives 1 mole of H _2_O). Therefore they can also be read as projections of the same straight line of slope μ (segment in the range 1<X<2 of the Rist diagram, Figure 2) ^9^.

This segment in the range 1<X<2 is particularly useful because it provides information on the average oxidation state of the reducing gas mixture (abscissa) and of the iron oxides (ordinate) ^12^. The abscissa can be interpreted as the reducing gas having an average oxidation state equal to X – 1 (see Eq.(26)). This means that at the abscissa X = 1, we find a reducing gas mixture composed by CO and H _2_, while at the abscissa X = 2 the gas is completely oxidized to CO _2_ and H _2_O. On the other hand, the Y coordinates represent the oxidation state of the iron oxides according to Eq.(27). This interpretation allow identifying the point A in the Rist diagram, whose ordinate is the initial oxidation state of the burden ( *e.g*., Y _A_ = 1.5 for Fe _2_O _3_) ^12^ and whose abscissa is the final degree of oxidation of the gas leaving the top of the furnace plus 1 ^13^.


$$\;\text{X}=1+\frac{{\text{CO}}_{\text{2}}+{\text{H}}_{2}\text{O}}{\text{CO}+{\text{CO}}_{\text{2}}+{\text{H}}_{2}+{\text{H}}_{2}\text{O}}\;(26)$$

$$\;\text{Y}=\frac{\text{O}}{\text{Fe}}\;(27)$$

The necessary condition to understand the segment 1<X<2 in this way is that the total number of moles of reducing gas keeps constant and that the oxygen supplied to the gas must come only from the reduction of the iron oxides ( *i.e*., no additional injections in the middle or upper zone, and no CaCO _3_ introduced, which would decompose into CaO and CO _2_ through calcination) ^12^. Moreover, it should be noted that such a correspondence between the abscissa and the composition of the mixture does not exist in the interval 0<X<1, where the segments could be arranged in any order.

### 3.3 Equation of the operating line

According to the theory described above, the equation of the operating line can be written as Eq.(28). The slope, μ, is the number of moles of reducing gas required for the production of 1 mole of Fe. The intercept, Y _E_, represents the moles of H _2_ and O coming from sources other than iron oxides that contribute to the formation of the reducing gas (negative sign by convention, Eq.(23)) ^12^.


$$\;\text{Y}=\text{μ}\cdot \text{X}+{\text{Y}}_{\text{E}}\;(28)$$

If the operating line is characterized, relevant information can be deduced from it. The slope accounts for the total reducing agent rate required (in terms of C and H _2_ per mole of Fe) so, if an auxiliary fuel is introduced, the decrease in the input rate of coke can be computed ^5^. The intercept stands for the hydrogen and oxygen brought into the furnace (except for the O _2_ contained in the iron ore), therefore the necessary air flow rate can be calculated by subtracting the other O _2_ and H _2_ sources (moisture, auxiliary fuels, coke and impurities) ^5^. Also, the initial oxidation state of the iron oxides introduced in the blast furnace ( *Y _A_*) allows to know the final degree of oxidation of the gas leaving the top of the furnace ( *X _A_* – 1). Finally, the ratio between direct and indirect reduction is identified by construction. The abscissa X = 1 gives the oxygen removed by direct reduction, *y _d_* ( Figure 2), and then the oxygen removed by indirect reduction is easily calculated as *y _i_* = *Y _A_* – *y _d_*.

### 3.4 Equilibrium line

In the blast furnace, the reducing gas can never oxidize the solids. For this reason, the operating line in the segment 1<X<2 must necessarily remain on the left of the equilibrium line of the Fe-O-H-C system ( Figure 2). If we were at some point at the right of the equilibrium line, the way to reach equilibrium would be displacing us upwards (i.e., providing O to the Fe) or leftwards (removing O from the gas), what in both cases means to oxidize the solids.

**Figure 3.**
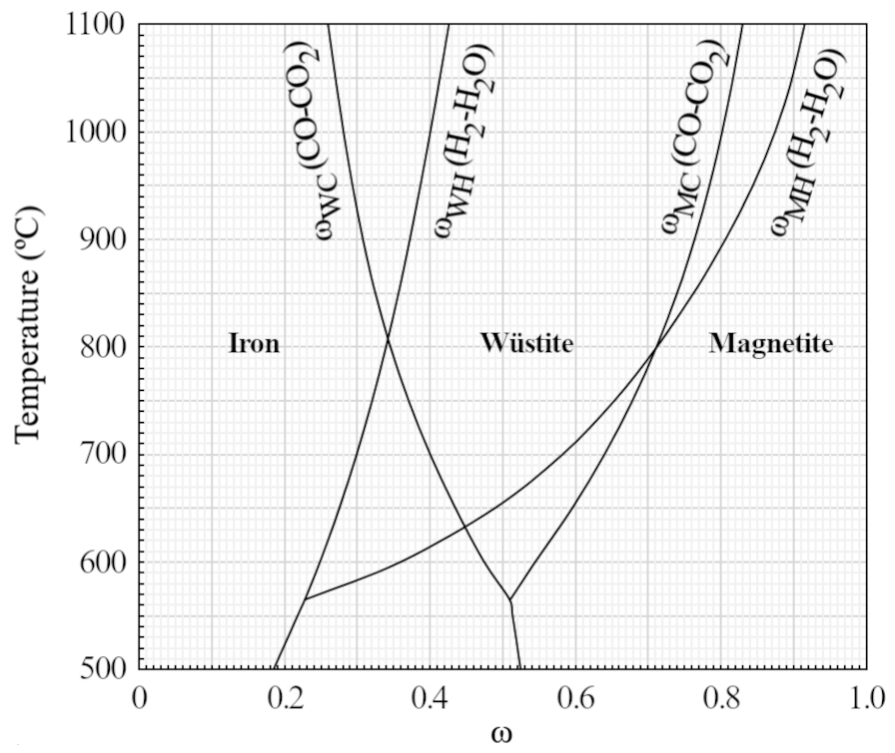
Equilibrium of the Fe-O-C and Fe-O-H systems. The variable ω stands for n _CO2_/(n _CO_+n _CO2_) in the case of *ω _WC_* and *ω _MC_*, and for n _H2O_/(n _H2_+n _H2O_) in the case of *ω _WH_* and *ω _MH_*.

The contour of the equilibrium line is delimited by five points, which we will denoted by F, W, W2, M, M2. The point W is of special interest since it corresponds to the chemical equilibrium between gases and solids at the beginning of the middle zone, where pure wüstite is found if the blast furnace operates under ideal conditions. The coordinates of these points are given in Table 1. The ordinates of the five points are easily calculated as the ratio of the oxygen and iron atoms of the corresponding components ( Eq.(27)) ^24^. The abscissae (X _w_ Eq.(29) and X _M_ Eq.(30), which are equivalent to Eq.(26)) depend on the molar fraction X _h_ that relates the hydrogen and water content of the reducing gas mixture ( Eq.(31)). This is used to combine the state of oxidation at equilibrium ω for the individual CO-CO _2_ and H _2_-H _2_O mixtures ( Figure 3). It should be noted that the molar fraction X _h_ is independent of the state of oxidation of the reducing gas ( *i.e.*, independent of the abscissa X), and can be calculated as a function of *y _e_*, *y _k_*, *a _j_* *y _j_* and *μ*.


$$\;{\text{X}}_{\text{W}}=1+(1-{\text{x}}_{\text{h}})\omega_{WC}+{\text{x}}_{\text{h}}\omega_{WH}\;(29)$$

$$\;{\text{X}}_{M}=1+(1-{\text{x}}_{\text{h}})\omega_{MC}+{\text{x}}_{\text{h}}\omega_{MH}\;(30)$$

$$\;x_{h}=\frac{{\text{H}}_{2}+{\text{H}}_{2}\text{O}}{\text{CO}+{\text{CO}}_{\text{2}}+{\text{H}}_{2}+{\text{H}}_{2}\text{O}}=\frac{\left(y_{e}+y_{k}+\sum a_{j}y_{j}\right)}{\mu }\;(31)$$

**Table 1.** Delimiting points of the equilibrium line for the Fe-O-H-C system in a Rist diagram.

		Abscissa X	Ordinate Y
F	Iron Fe	X _F_=X _W_	Y _F_=0
W	Wüstite Fe _0.95_O	X _W_= Eq.(29)	Y _W_=1.05
W2	Wüstite Fe _0.89_O	X _W2_=X _M_	Y _W2_=1.12
M	Magnetite Fe _3_O _4_	X _M_= Eq.(30)	Y _M_=1.33
M2	Magnetite Fe _3_O _4_	X _M2_=2	Y _M2_=1.33

For computing *ω*, Chaudron diagrams or tabulated data must be used ^9^. In our case, we adjusted the data from 10, 23 to a polynomic equation where *T* is given in °C ( Eq.(32)).


$$\;\omega =a_{0}+a_{1}T+a_{2}T^{2}+a_{3}T^{3}+a_{4}T^{4}+a_{5}T^{5}\;(32)$$

The coefficients are presented in Table 2. The temperature at which *ω* is calculated corresponds to the temperature of the middle zone (normally between 800 and 1000°C), where the chemical equilibrium between the gas and the solids occurs. We denote this temperature as *T _R_*.

**Table 2.** Coefficients for the calculation of the oxidation state at equilibrium in Fe-O-C and Fe-O-H systems ( Eq.(32)). Valid from 500 to 1100°C.

	T (°C)	Equilibrium	*a*_0_·10	*a*_1_·10 ^3^	*a*_2_·10 ^5^	*a*_3_·10 ^8^	*a*_4_·10 ^11^	*a*_5_·10 ^15^
*ω _WC_*	>565	Iron-Wüstite	4.0894101	3.8856440	-1.3778206	1.7924558	-1.0465659	2.3054702
	<565	Iron-Magnetite	6.3672662	-0.2230216	0	0	0	0
*ω _MC_*	>565	Wüstite-Magnetite	-7.8765294	3.6286637	-0.2811151	0.0772069	0	0
	<565	Iron-Magnetite	6.3672662	-0.2230216	0	0	0	0
*ω _WH_*	>565	Iron-Wüstite	-0.4957779	-0.5074581	0.4367340	-0.6744924	0.4402173	-1.0668513
	<565	Iron-Magnetite	-1.4558633	0.6611511	0	0	0	0
*ω _MH_*	>565	Wüstite-Magnetite	-215.36181	115.54294	-24.611536	26.555149	-14.341369	30.845286
	<565	Iron-Magnetite	-1.4558633	0.6611511	0	0	0	0

## 4. Calculation of the operating line

In practice, and especially when predicting new operating conditions, the equation of the operating line cannot be directly computed by calculating μ and Y _E_ because the required data is missing. The operating line must be obtained through two characteristic points denoted as R (coordinates X _R_, Y _R_) and P (coordinates X _P_, Y _P_). The former represents the equilibrium between gases and solids reached in the middle zone. The latter is a fixed point imposed by the energy balance of the blast furnace. When these two points of the operating line are known, it is easy to compute the slope and the Y-intercept of the operating line ( Eq.(33) and Eq.(34)):


$$\;\text{μ}=\frac{{\text{Y}}_{\text{R}}-{\text{Y}}_{\text{P}}}{{\text{X}}_{\text{R}}-{\text{X}}_{\text{P}}}\;(33)$$

$$\;{\text{Y}}_{\text{E}}={\text{Y}}_{\text{R}}-{\text{X}}_{\text{R}}\left(\frac{{\text{Y}}_{\text{R}}-{\text{Y}}_{\text{P}}}{{\text{X}}_{\text{R}}-{\text{X}}_{\text{P}}}\right)\;(34)$$

In the following subsections, it is explained how to calculate the points R and P. The additions and corrections to the original work of Rist are introduced mainly during the calculation of the energy balance (explained more in detail in the appendixes ^25^). These are the following:

▪      All calculations are given as a function of the temperature of the middle zone, *T _R_* ( *i.e.*, of the temperature of the thermal reserve zone where thermal equilibrium exists).

▪      Each auxiliary fuel can enter at different temperatures because they are now treated separately, rather than as an overall injection.

▪      The heat associated to the direct reduction of FeO is now corrected taking into account the moisture of the hot blast.

▪      The sensible heat of the hot metal is calculated as a function of its composition.

▪      The sensible heat of the slag is calculated as a function of its SiO _2_, Al _2_O _3_, CaO and MgO content.

▪      The heat of carburization is calculated as a function of the austenite and cementite content in iron.

▪      The heat associated to the lack of thermal ideality is calculated as a function of the burden and coke composition.

▪      The heat associated to the lack of chemical ideality is calculated as a function of the burden in the thermal reserve zone.

▪      The heat associated to the lack of chemical ideality now accounts also for the hydrogen coming from auxiliary fuels and for the moisture of the hot blast.

▪      A supplementary model is added to compute the heat of decomposition of coal when injected as auxiliary fuel.

▪      An additional energy balance in the upper zone of the blast furnace is added to compute the final composition of the blast furnace gas, instead of only computing the final oxidation state.

### 4.1 Point W and point R: chemical and thermal reserve zones

In an ideal blast furnace, the reducing gas and the burden are in chemical equilibrium after the upper zone. This is known as the chemical reserve zone, in which wüstite is the only iron oxide present. This point is denoted by W and was already identified during the construction of the equilibrium line ( Table 1).

In practice, a blast furnace does not operate under ideal conditions, so a zone of pure wüstite cannot be distinguished (the operating line no longer passes through the point W). For these cases, it is defined the chemical efficiency of the furnace, *r*, representing the oxygen actually exchanged to the oxygen theoretically exchangeable (typically, *r* is around 0.92) ^5, 10^. The coordinates of the new point R, through which the operation line passes, is calculated by Eq.(35) and Eq.(36) as a function of the point W, the chemical efficiency *r*, and the initial oxidation state of the burden Y _A_. ^10^.


$$\;{\text{X}}_{\text{R}}=1+r({\text{X}}_{\text{W}}-1)\;(35)$$

$$\;{\text{Y}}_{\text{R}}={\text{Y}}_{\text{A}}-r({\text{Y}}_{\text{A}}-{\text{Y}}_{\text{W}})\;(36)$$

Despite there is no chemical equilibrium, the temperature is still nearly constant in the middle zone (thermal reserve zone where thermal equilibrium exists), so this temperature is used for the calculations ( *T _R_*).

### 4.2 Point P: energy balance in the elaboration zone

The operating line, Eq.(28), depends on different parameters which are not all independent. In particular, any energy balance, whether global or partial, imposes a relationship between them. An option is to use the energy balance of the elaboration zone ( *i.e.*, middle zone plus lower zone), which follows Eq.(37). As in the original work of Rist, we are going to work in kcal per mole of Fe ^10^. Moreover, the reference temperature for the energy balance is chosen at *T _R_*, *i.e.*, at the temperature of the thermal reserve zone.


$$q_{c}y_{v}+q_{v}y_{v}+(q_{iw}{\text{Y}}_{\text{W}}-\delta )=q_{g}(y_{d}-y_{e})+q_{k}y_{k}+q_{e}y_{e}+\sum q_{j}y_{j}-\delta^{\prime }+q_{\text{Si}}y_{\text{Si}}+q_{\text{Mn}}y_{\text{Mn}}+q_{P}y_{P}+q_{\gamma }\gamma +f+l+p+C_{\text{Δ}T_{R}}\;(37)$$

The input/production of energy is on the left side of the equation, and the output/consumption is on the right side of the equation. Regarding the former, we have the term *q _c_* *y _v_* that represents the heat released by the incomplete combustion of carbon, *q _v_* *y _v_* for the sensible heat of the air, *q _iw_* *Y _w_* as the heat released by the reduction of wüstite, and *δ* to account for the lack of chemical ideality in wüstite reduction. In the right side of the equation we have *q _g_* ( *y _d_* – *y _e_*) which is the heat absorbed during direct reduction because of CO _2_ dissociation, *q _k_* *y _k_* as the heat consumed due to the hydrogen entering with the coke, *q _e_* *y _e_* for the overall heat absorbed by the moisture of the air, $\sum q_{j}y_{j}$ to quantify the overall heat absorbed by the auxiliary injections, $\delta^{\prime }$ accounting for the lack of chemical ideality in the conversion of H _2_ to H _2_O, ( *q*_Si_ *y*_Si_ + *q*_Mn_ *y*_Mn_ + *q _P_* *y _P_*) for the heat absorbed by the reduction of the accompanying elements SiO _2_, MnO and P _2_O _5_, then *q _γ_* *γ* as the heat absorbed by the carburization of the iron, *f* denoting the heating and melting of the hot metal, *l* for the heating and melting of the slag, *p* for the heat removed by the staves and, lastly, ( *C*_Δ *T*_*R*__) accounting for the lack of thermal ideality (in case the temperature of the gas and the solid is not the same at the thermal reserve zone).

All the addends of Eq.(37) are thoroughly explained in the Appendix A ^25^ to avoid breaking here the flow of the explanation. Besides, they are compared with the calculation method appearing in Rist’s original work when appropriate to highlight the differences. The meaning and units of each variable is specified in the nomenclature list, and a table summarizing the calculation of the heats denoted by *q* is available in Table 6 of Appendix A ^25^.

From the energy balance of Eq.(37), we can find a relation between *y _d_* (number of O moles removed from iron oxides by direct reduction) and *y _v_* (number of O moles brought by the air), under given operating conditions defined by the inlet/outlet temperatures, the chemical efficiency, the tuyeres injections, and the composition of hot metal and slag. This relation can be written as Eq.(38), where A, B and C are given by Eq.(39), Eq.(40) and Eq.(41).


$$\;y_{d}=\frac{Ay_{v}-\text{C}}{\text{B}}\;(38)$$

$$\;\text{A}=q_{c}+q_{v}-eq_{e}+eq_{k}(1-r)+eq_{g}\;(39)$$

$$\;\text{B}=q_{g}\;(40)$$

$$\text{C}=-q_{iw}Y_{w}+\delta +q_{k}y_{k}+\sum q_{j}y_{j}-(1-r)\left(y_{k}+\sum a_{j}y_{j}\right)q_{k}+q_{\text{Si}}y_{\text{Si}}+q_{\text{Mn}}y_{\text{Mn}}+q_{P}y_{P}+q_{\gamma }\gamma +f+l+p+C_{\text{Δ}T_{R}}\;(41)$$

Here, the variable *e* denotes the moles of H _2_O per O moles in the air ( *i.e.*, *e* = *y _e_*/ *y _v_*), which is a convenient notation to write the water injected with the air as a function of the air injected through the tuyeres ( *i.e.*, to include the term *q _e_* *y _e_* in A). Similarly, the term $\delta^{\prime }$ was decomposed according to Eq.(92) to separate the terms that depends on *y _v_* (see Appendix A.9 ^25^). It should be noted that Rist did not decompose $\delta^{\prime }$ and therefore he wrongly included a term that is actually dependent on *y _v_* in C instead of adding it to A ^10^. Moreover, Rist did not included the term + *eq _g_* that appears in A, which corrects the heat absorbed during direct reduction (part of the direct reduction takes place through Eq.(10), see Appendix A.5).

Now, we can impose the relation of Eq.(38) from the energy balance to the operating line defined by Eq.(28). To do that, we first rewrite Eq.(28) as Eq.(42), taking into account that μ = –Y _E_ + *y _d_* according to Eq.(22) and Eq.(23).


$$\;\text{Y}=-{\text{Y}}_{E}(\text{X}-1)+y_{d}\text{X}\;(42)$$

By substituting Eq.(38) in Eq.(42), and by using the convenient notation of Eq.(43) to decompose Y _E_ on two terms (one dependent of *y _v_* and another independent), we found Eq.(44) where Δ _1_ and Δ _2_ are given by Eq.(45) and Eq.(46).


$$\;{\text{Y}}_{E}^{*}\equiv {\text{Y}}_{E}+(1-2e)y_{v}=-\sum (a_{j}+2b_{j})y_{j}-y_{si}-y_{Mn}-y_{P}-y_{S}-y_{k}\;(43)$$

$$\;\Delta_{1}y_{v}+\Delta_{2}=0\;(44)$$

$$\;\Delta_{1}=\left(\frac{\text{A}}{B}+1+2\text{e}\right)\text{X}-(1+2\text{e})\;(45)$$

$$\;\Delta_{2}=-\text{Y}-\left(\frac{\text{C}}{B}+{\text{Y}}_{E}^{*}\right)\text{X}+{\text{Y}}_{E}^{*}\;(46)$$

From this operating line in the form of Eq.(44), which accounts for the energy balance, we know that the operating line will pass through a point *P* of coordinates X _p_ and Y _p_ fulfilling simultaneously Δ _1_ = 0 and Δ _2_ = 0. Applying this condition, we can find from Eq.(45) and Eq.(46) the coordinates X _p_ and Y _p_ ( Eq.(47) and Eq.(48)). Now, the operating line of the blast furnace is known.


$$\;{\text{X}}_{P}=\frac{\text{B}(1+2\text{e})}{\text{A}+\text{B}(1+2\text{e})}\;(47)$$

$$\;{\text{Y}}_{P}={\text{Y}}_{E}^{*}-\left(\frac{\text{C}}{B}+{\text{Y}}_{E}^{*}\right)\left(\frac{\text{B}(1+2\text{e})}{\text{A}+\text{B}(1+2\text{e})}\right)\;(48)$$

It is worth to mention that both coordinates of *P* depend on the chemical efficiency of the furnace, *r*, through A and C. However, Rist only considered the dependence on the chemical efficiency through C because he did not decompose $\delta^{\prime }$ . Therefore, he found that X _p_ was independent of *r* ^10^. Here we have shown that is not.

### 4.3 Additional results derived from the operating line

The relevance of characterizing the operating line comes from the possibility of deducing operational data such as the required reducing agent rate, the air consumption, the top gas composition, the ratio between direct and indirect reduction, and the flame temperature.

***4.3.1 Reducing agent rate (coke consumption).*** The reducing agent rate, μ, obtained from the operating line, denotes the number of moles of C and H _2_ needed inside the blast furnace as reducing gas for the production of 1 mole of Fe as hot metal. When solving the Rist diagram, we assume the auxiliary injections to be known. Therefore, we can compute the required amount of coke by subtracting the contributions of the injections to μ. The carbon that ends up dissolved in the hot metal must be also taken into account (which increases the required reducing agent rate), as well as the H _2_ that is produced when the H _2_O from the hot blast is dissociated (which decreases the required reducing agent rate). Thus, the mass flow of coke is calculated by Eq.(49).


$$\;{\text{m}}_{K}=(\mu \;+\gamma \;-ey_{v}-\sum (\tau_{j}+a_{j})y_{j})n_{\text{HM},\text{Fe}}\text{/}{}_{coke}\;(49)$$

In this equation, *γ* is the number of moles of C dissolved in the hot metal ( Eq.(50)), *τ _j_* is equal to 1 when the auxiliary injection contains carbon and 0 when not, and _K_ is the ratio of C and H _2_ moles in coke per kg of coke ( Eq.(51)). The variables Ω *_j,i_* are the mass fraction of element *i* in compound *j*.


$$\;\gamma =\left(10^{6}\Omega_{\text{HM},\text{C}}/{\text{M}}_{C}\right)/n_{\text{HM},\text{Fe}}\;(50)$$

$$\;{}_{\text{K}}=10^{3}\left(\frac{\Omega_{\text{K},\text{C}}}{{\text{M}}_{C}}+\frac{\Omega_{\text{K},\text{H}}}{{\text{M}}_{\text{H}2}}\right)\;(51)$$

The term *n*_HM,Fe_ is the number of moles of Fe in hot metal per ton of hot metal (Eq.(74)).

***4.3.2 Air flow rate.*** The intercept of the operation line, denoted by Y _E_, represents the H _2_ and O brought into the furnace (except for the O contained in the iron ore) with negative sign by convention. By subtracting the O and H _2_ sources other than the hot blast, the necessary O flow rate as air can be calculated ( *i.e.*, calculation of *y _v_*, Eq.(52))


$$\;y_{v}=\frac{-(Y_{E}+\sum (a_{j}+2b_{j})y_{j}+y_{Si}+y_{Mn}+y_{P}+y_{S}+y_{k})}{1+2e}\;(52)$$

Once we know *y _v_*, the mass of air (dry) per ton of hot metal is calculated by Eq.(53).


$$\;m_{v}^{d}=\frac{y_{v}n_{\text{HM},\text{Fe}}}{2000}\left({\text{M}}_{\text{O}2}+\frac{0.79}{0.21}{\text{M}}_{\text{N}2}\right)\;(53)$$

***4.3.3 Blast furnace gas composition.*** Once the coke and air flow rate are known, the input streams to the blast furnace become completely characterized. In order to characterize also the output streams, we have to find 15 unknown variables. These are the mass flow rate of each component in the blast furnace gas ( *m*_BFG,CO_, *m*_BFG,CO2_, *m*_BFG,H2_, *m*_BFG,H2O_, *m*_BFG,N2_), the mass flow rate of hot metal *m*_HM_ (the individual mass flow rate of each component in hot metal is calculated through its mass fraction, which is assumed known from the beginning), and the mass flow rate of each component in the slag ( *m*_Slag,SiO2_, *m*_Slag,Al2O3_, *m*_Slag,CaO_, *m*_Slag,MgO_, *m*_Slag,MnO_, *m*_Slag,CaS_, *m*_Slag,P2O5_, *m*_Slag,Fe2O3_, *m*_Slag,FeO_). The system of 15 equations to solve the balance comprises 11 mole balances for Fe, Si, Al, Ca, Mg, Mn, C, H, N, S, P (the O balance was already accounted in the elaboration of the operating line), two mass balances for *m*_Slag,Fe2O3_, *m*_Slag,FeO_ (they correspond to the Fe _2_O _3_ and FeO content of the coal ashes, which enter at the tuyeres and are not reduced), one equation related to the final oxidation state of the blast furnace gas (information coming from the operating line, Eq.(54)), and one energy balance of the preparation zone ( *i.e.*, of the upper zone).


$$\;\eta_{\text{CO}-{\text{H}}_{2}}\equiv \frac{n_{\text{BFG},\text{C}{\text{O}}_{2}}+{\text{n}}_{\text{BFG},{\text{H}}_{2}\text{O}}}{{\text{n}}_{\text{BFG},\text{CO}}+{\text{n}}_{\text{BFG},\text{C}{\text{O}}_{2}}+{\text{n}}_{\text{BFG},{\text{H}}_{2}}+{\text{n}}_{\text{BFG},{\text{H}}_{2}\text{O}}}=\frac{{\text{Y}}_{A}-{\text{Y}}_{E}}{\text{μ}}-1\;(54)$$

It should be noted that knowing the final oxidation state of the gas exiting the top of the furnace ( *i.e.*, Eq.(54)) is not sufficient to compute the final composition of the top gas because the gas is not in equilibrium at the upper part of the furnace. In other words, the water–gas shift reaction ( Eq.(7)) changes the BFG composition without modifying *η*_CO–H2_, but the extent of this reaction is unknown. For this reason, the energy balance of the preparation zone is required. This energy balance is detailed in Appendix C ^25^.

Most authors use the CO utilization ratio ( *η*_CO_ ≡ *n*_BFG_, _CO _2__/(n _BFG_, _CO_ + n _BFG,CO _2__)) as the 15 ^th^ equation to complete the system of equations, instead of using the energy balance in the upper zone. They assume to know this parameter, since in practice they would be able to measure it at the flue gas of a real ironmaking plant. Indeed, in those cases in which the operating line is characterized for a real operation where the *η*_CO_ information is reliable, to use this procedure instead of the energy balance of the upper part makes the methodology easier. However, this is not a valid procedure when analyzing new operating lines for potential blast furnace configurations. In such case, the assumption of the value of *η*_CO_ would be arbitrary and, almost certainly, it will lead to inconsistencies in the upper zone (energy balance not fulfilled). Inconsistencies which are not detected because of not performing the corresponding energy balance in the upper zone. For this reason, it is completely necessary to use the energy balance of the upper zone instead of *η*_CO_ when proposing new operating lines.

***4.3.4 Amount of direct and indirect reduction.*** By construction, the abscissa X = 1 allows computing *y _d_* ( Figure 2), which is the oxygen removed from the iron oxides by direct reduction ( Eq.(55)).


$$\;y_{d}=\mu +{\text{Y}}_{E}\;(55)$$

Then, the oxygen removed by indirect reduction is easily calculated as Eq.(56).


$$\;y_{i}={\text{Y}}_{A}-y_{d}\;(56)$$

***4.3.5 Flame temperature.*** The flame temperature is the temperature that the raceway gas reaches when all oxygen from hot blast and injections has been used for the incomplete combustion of C to CO, and all water has been dissociated to CO and H _2_. It can be considered as a control parameter to check if the studied configuration of blast furnace is reasonable, since the injection of auxiliary fuels drops the flame temperature as a consequence of their lower C/H ratio compared with coke (flame temperature should be kept between 2000 and 2300°C for a proper operation). In such cases, blast oxygen enrichment may be required to maintain a suitable flame temperature.

From a theoretical point of view, this flame temperature can be calculated from an energy balance in the raceways. We use Eq.(57), where we made a similar construction to that of Eq.(37). The term *q _c_* *y _v_* stands for the combustion of C to CO by using the O from the hot blast, the term *q _v_* *y _v_* represents the sensible heat that the dry hot blast is providing, while the term q _*s*,C_y _C_ is the sensible heat of the coke carbon used in combustion (which comes at *T _f_* from the lower zone). At the right side of the equation, we have ( *q _er_* + *q _es_*) *y _e_* which is the contribution of the moisture of the hot blast (decomposition and sensible heat), also $\sum_{j}\left(q_{jd}+q_{js}-2b_{j}q_{c}\right)y_{j}$ for the auxiliary injections (decomposition, sensible heat and combustion of the O entering with this injections), and *q*_*s*,N2_ *y*_rg,N2_ + *q*_*s*,CO_ *y*_rg,CO_ + *q*_*s*,H2_ *y*_rg,H2_, as the energy required to heat the reducing gas up to the temperature of the flame *T _fl_*. Lastly, it is included the heating from *T _R_* to *T _fl_* of the ashes from coal injections $\left(\sum_{j}Z_{j}q_{s,\text{Z}}y_{j}\right).$


$$\;q_{c}y_{v}+q_{v}y_{v}+q_{s,C}y_{C}=(q_{er}+q_{es})y_{e}+\sum_{j}(q_{jd}+q_{js}-2b_{j}q_{c})y_{j}+\sum_{i}q_{s,i}y_{\text{rg},i}+\sum_{j}z_{j}q_{s,\text{Z}}y_{j}\;(57)$$

We can say that the flame temperature is a result indirectly derived from the operating line since for its computation we need to know the amount of air injected, the amount of coke, and the amount and composition of the reducing gas (obtained from the composition of the BFG). The variables *y _v_*, *y _C_*, *y*_*rg*,N2_, *y*_*rg*,CO_ and *y*_*rg*,H2_ are calculated by Eq.(52), and Eq.(58) to Eq.(61). In the case of *y _C_*, we can compute it as the oxygen available for combustion minus the C coming from the injections (because 1 mole of O consumes 1 mole of C), or as the C coming from coke minus the C used in reactions other than combustion. The variable *τ _j_* is equal to 1 when the auxiliary injection contains carbon and 0 when not.


$$\;y_{\text{C}}=y_{v}+\sum_{j}\left(2b_{j}-\tau_{j})y_{j}=\frac{10^{3}\Omega_{\text{K},\text{C}}{\text{m}}_{K}}{{\text{M}}_{C}n_{\text{HM},\text{Fe}}}-(\gamma +y_{Si}+y_{Mn}+y_{P}+y_{S}+y_{d}\right)\;(58)$$

$$\;y_{\text{rg},\text{N}2}={{\text{n}}_{\text{BFG},{\text{N}}_{2}}/n}_{\text{HM},\text{Fe}}\;(59)$$

$$\;y_{\text{rg},\text{CO}}=({{\text{n}}_{\text{BFG},\text{CO}}+{\text{n}}_{\text{BFG},\text{C}{\text{O}}_{2}})/n}_{\text{HM},\text{Fe}}\;(60)$$

$$\;y_{\text{rg},{\text{H}}_{2}}=({{\text{n}}_{\text{BFG},{\text{H}}_{2}}+{\text{n}}_{\text{BFG},{\text{H}}_{2}\text{O}})/n}_{\text{HM},\text{Fe}}\;(61)$$

The calculation of the rest of the terms were already explained in Appendix A ^25^ for the energy balance of the elaboration zone. All the *q* terms are tabulated in Table 6 according to the adjustment to Eq.(113) or Eq.(114). The variable T in these equations must be substituted by *T _R_* for *q _c_* and *q _er_*, by *T _v_* for *q _v_* and *q _es_*, by *T _f_* for *q*_*s*,C_, by *T _j_* for *q _jd_* and *q _js_*, and by *T*_fl_ for *q _s,i_* and *q*_*s*,Z_. Thus, the only missing variable in Eq.(57) is *T*_fl_.

In practice, different authors have developed formulae for the calculation of the flame temperature as a function of different operating parameters ^16^. Here we present the equation developed by Babich ^16, 26^, which accounts for natural gas and pulverized coal injections ( Eq.(62)). We will use it for comparison and validation purposes.


$${\text{T}}_{\text{fl}}=\frac{44.455V_{v}T_{v}(c_{p,v}+0.0012c_{p,\text{H}2\text{O}}\eta_{nat})+3146V_{v}\omega_{v,{\text{O}}_{2}}-1170V_{NG}\omega_{NG,\text{CH}4}-600m_{PC}\Omega_{P\text{C},C}-1.65V_{v}\eta }{(0.2387V_{v}+0.24V_{v}\omega_{v,{\text{O}}_{2}}+0.48V_{NG}\omega_{NG,\text{CH}4}+6m_{PC}\Omega_{PC,\text{H}}+0.0006V_{v}\eta )186.785c_{p,rg}}\;(62)$$

Where *V _v_* is the dry blast volume per ton of hot metal, *T _v_* is the temperature of the blast, *ω*_*v*,O _2__ is the oxygen content in the blast, *η _nat_* is the natural moisture in the blast, *η* is the total moisture in the blast, *V _NG_* is the natural gas consumption, *m _PC_* is the pulverized coal consumption, Ω _*PC*,C_ is the carbon content in pulverized coal, Ω _*PC*,H_ is the hydrogen content in pulverized coal, *ω*_*NG*,CH4_ is the methane content in natural gas, and *C _p,v_*, *C*_*P*,H2O_ and *C _p,rg_* are the specific heats of blast, moisture and reducing gas. The units are provided in the nomenclature list.

## 5. Model validation

**Figure 4.**
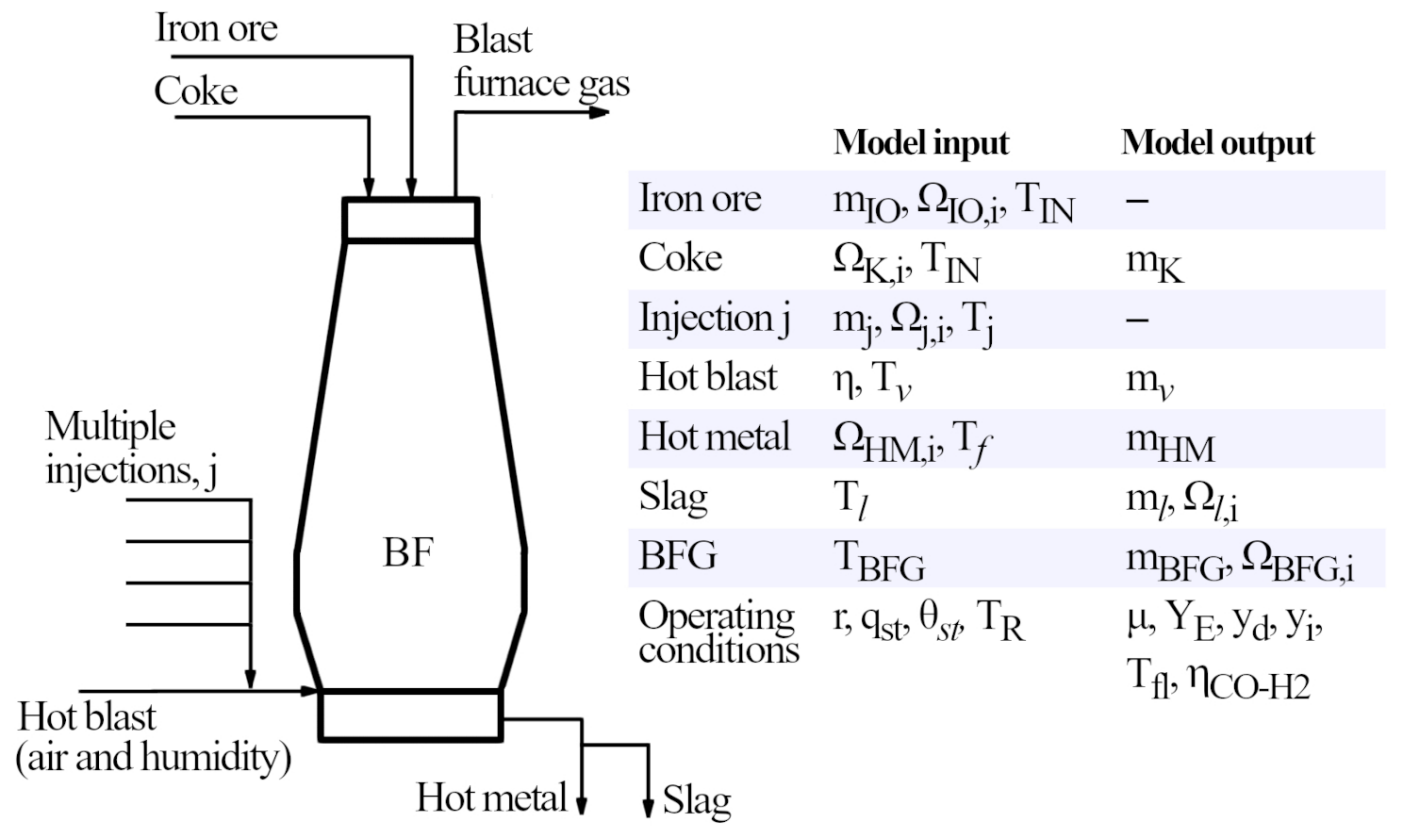
Conceptual diagram of a blast furnace, indicating the input and output data of the model.

In order to validate the model, we are going to use three different data sets. The first one comes from the original work of Rist ^10^, corresponding to a conventional air-blown blast furnace without auxiliary injections. The second one is elaborated from the work of Babich *et al.* ^18^, for an air-blown blast furnace with pulverized coal injection and O _2_-enriched air. The last one is taken from the work of Sahu *et al.* ^27^ for an oxygen blast furnace with top gas recycling. When solving the operating diagram, we assume the composition of iron ore, coke, auxiliary injections and hot metal to be known. For iron ore and injections, the total mass flow is also known. For the hot blast, the moisture is fixed. Regarding energy balances, all the inlet and outlet temperatures are known. Finally, the operating conditions on chemical efficiency, heat removed by the staves, the proportion of heat evacuated between the preparation and elaboration zone, and the temperature of the thermal reserve zone are set to known values. Under this framework, the model allows to compute the mass flows of coke, hot blast, hot metal and blast furnace gas. Moreover, the composition of the blast furnace gas and slag can be calculated. Regarding operation conditions, the slope and intercept of the operating line, the amount of direct and indirect reduction, the flame temperature and the oxidation state of the BFG are obtained. The list of inputs and outputs are summarized in Figure 4 together with a conceptual scheme of the blast furnace. Exceptionally, for those cases in which we are not introducing air in the blast furnace, we assume the mass of the host blast to be known ( *m _v_* = 0) and we calculate instead the mass flow of the injection that carries the main oxygen input ( *m _j_*).

### 5.1 Air-blown blast furnace without auxiliary injections (Rist, Data set 1)

This data set was elaborated from the original work of Rist ^10^, corresponding to an air-blown blast furnace without auxiliary injections. The input mass flow of iron ore and its composition was calculated through mass balances based on Rist’s results, assuming typical mass fractions for those oxides other than iron. Also, it was assumed a typical mass fraction distribution for oxides in coke ^28^. The outlet temperature of BFG was assumed to be 180°C to estimate the gas composition.

As explained through the model description and appendixes ^25^, three potential errors were identified in the original model provided by Rist: (1) underestimation of the heat required inside the blast furnace by not accounting for the heating of slag between *T _R_* and *T _f_* (see Appendix A.12 ^25^), (2) overestimation of the heat required because of wrongly computing the term *l* with *C*_*p*,SiO _2__ in wrong units (see Appendix A.13 ^25^) and (3) overestimation of the heat required because of considering twice the heat absorbed during direct reduction for a number of moles equals to *Y _e_* (see Appendix A.5 ^25^). For the sake of comparison, we intentionally introduced the same three errors in our model (third column in Table 3). This way, the model reproduces the results of Rist with a discrepancy below 3.5%, thus validating the consistency of the model.

**Table 3.** Comparison of model results with literature data sets for air-blown blast furnaces, for validation purposes. *This column shows the results of the model when corrections are not included (see text).

	DATA SET 1					DATA SET 2				
Input streams (kg/t _HM_)	Rist ^10^	Model *	Δ (%)	Model	Δ (%)	Babich ^18^	Model	Δ (%)
**Iron ore** (25°C)	**2276.4**	**2276.4**	**-**	**2276.4**	**-**	**1558.0**	**1558.0**	**-**
# Fe _2_O _3_	947.4	947.4	-	947.4	-	1146.9	1146.9	-
# FeO	357.3	357.3	-	357.3	-	187.3	187.3	-
# SiO _2_	327.5	327.5	-	327.5	-	69.9	69.9	-
# Al _2_O _3_	133.1	133.1	-	133.1	-	107.2	107.2	-
# CaO	446.0	446.0	-	446.0	-	26.9	26.9	-
# MgO	4.0	4.0	-	4.0	-	16.2	16.2	-
# MnO	19.9	19.9	-	19.9	-	3.6	3.6	-
# P _2_O _5_	41.2	41.2	-	41.2	-	0.0	0.0	-
**Coke** (25°C)	**598.9**	**612.8**	**2.3**	**619.9**	**3.5**	**283.0**	**289.0**	**2.1**
# C	509.0	520.9	2.3	526.9	3.5	251.4	256.7	2.1
# H	3.0	3.1	2.3	3.1	3.3	-	-	-
# S	8.2	8.4	2.3	8.5	3.6	-	-	-
# Fe _2_O _3_	-	-	-	-	-	1.7	1.8	2.1
# SiO _2_	26.5	27.1	2.3	27.4	3.5	19.7	20.1	2.1
# Al _2_O _3_	10.8	11.0	2.3	11.2	3.5	9.1	9.3	2.1
# CaO	36.1	37.0	2.3	37.4	3.5	0.8	0.8	2.1
# MgO	5.4	5.5	2.2	5.5	3.6	0.3	0.3	2.1
**Coal** (25°C)	-	-	-	-	-	**200.0**	**200.0**	**-**
# C	-	-	-	-	-	153.6	153.6	-
# H	-	-	-	-	-	8.3	8.3	-
# O	-	-	-	-	-	10.2	10.2	-
# N	-	-	-	-	-	3.1	3.1	-
# S	-	-	-	-	-	0.9	0.9	-
# H _2_O	-	-	-	-	-	2.4	2.4	-
# SiO _2_	-	-	-	-	-	12.3	12.3	-
# Al _2_O _3_	-	-	-	-	-	8.9	8.9	-
# CaO	-	-	-	-	-	0.3	0.3	-
**Hot blast** (700°C Rist / 1200°C Babich)	**2125.6**	**2198.7**	**3.4**	**2242.5**	**5.5**	**1077.8**	**1086.6**	**0.8**
# N _2_	1618.0	1673.6	3.4	1706.9	5.5	826.7	833.5	0.8
# O _2_	491.0	508.2	3.5	518.3	5.6	251.1	253.1	0.8
# H _2_O	16.6	16.9	2.0	17.3	4.1	0.0	0.0	-
**O _2_ enrichment** (1200°C)	**-**	**-**	**-**	**-**	**-**	**94.3**	**95.1**	**0.8**
Output streams (kg/t _HM_)								
**Hot metal** (1400°C Rist / 1500°C Babich)	**1000.0**	**1000.0**	**0.0**	**1000.0**	**0.0**	**1000.0**	**1000.0**	**0.0**
# Fe	937.0	937.0	0.0	937.0	0.0	947.2	947.2	0.0
# C	38.0	38.0	0.0	38.0	0.0	45.0	45.0	0.0
# Si	4.0	4.0	0.0	4.0	0.0	5.3	5.3	0.0
# Mn	3.0	3.0	0.0	3.0	0.0	2.5	2.5	0.0
# P	18.0	18.0	0.0	18.0	0.0	0.0	0.0	0.0
**Slag** (1450°C Rist / 1550°C Babich)	**1000.0**	**1002.8**	**0.3**	**1003.8**	**0.4**	**260.0**	**261.8**	**0.7**
# SiO _2_	-	346.1	-	346.4	-	90.4	91.0	0.7
# Al _2_O _3_	-	144.1	-	144.3	-	124.6	125.4	0.7
# CaO	-	468.4	-	468.6	-	26.3	26.5	0.7
# MgO	-	9.5	-	9.5	-	16.4	16.5	0.7
# MnO	-	16.0	-	16.0	-	0.4	0.4	0.7
# CaS	-	18.8	-	19.0	-	1.9	1.9	0.7
# P _2_O _5_	-	0.0	-	0.0	-	-	-	-
**BFG** (180°C Rist / 150°C Babich)	**3000.0**	**3085.2**	**2.8**	**3135.1**	**4.5**	**1953.2**	**1966.9**	**0.7**
# N _2_	-	1673.6	-	1706.9	-	830.0	836.6	0.8
# CO _2_	-	743.1	-	735.4	-	678.8	684.4	0.8
# CO	-	653.1	-	672.0	-	414.7	416.2	0.4
# H _2_O	-	11.8	-	17.7	-	23.9	23.8	-0.6
# H _2_	-	3.6	-	3.0	-	5.9	5.9	0.0
Operating conditions								
Chemical efficiency, *r* (-)	1.000	1.000	-	1.000	-	-	0.92	-
Thermal reserve zone temperature, T _R_ (°C)	1000.0	1000.0	-	1000.0	-	-	800	-
Flame temperature, T _fl_ (°C)	-	1879.0	-	1881.0	-	2117.0	2138.6	1.0
Heat evacuated by the staves (MJ/THM)	418.4	418.4	-	418.4	-	701.1	701.1	-
Heat evacuated in the elaboration zone, *θ*_st_ (-)	0.700	0.700	-	0.700	-	-	0.800	-
Oxidation state of the BFG, η _CO–H2_ (-)	-	0.411	-	0.410	-	0.486	0.487	0.2

The results of the model are also presented with the three mentioned corrections included (fifth column in Table 3). In this case, the variation with respect to the data provided by Rist is beyond 5% in some cases, without overpassing the 6%. Fortunately for Rist, the three errors counterbalanced, and reasonable results could be achieved despite of them. Making the comparison fair, the variation between the results of our model with and without the corrections is in the range 1–2%. This clearly shows how well the three errors counterbalanced.

Looking for further validation, we compare the flame temperature computed through our model with the calculated by Eq.(62). The latter gives 1898°C (see Appendix D ^25^) and we obtained 1879°C with our model, what means we have a discrepancy of only 1.0%. In both cases, the flame temperature is below 2000°C, which is not suitable for blast furnaces. Nevertheless, the purpose of this data set is validation only, which can be assumed achieved. The type of configuration provided by this data set does not correspond to a state-of-the-art blast furnace, since nowadays most blast furnaces use auxiliary injections like pulverized coal and burdens of greater quality that lead to lower slag productions.

**Figure 5.**
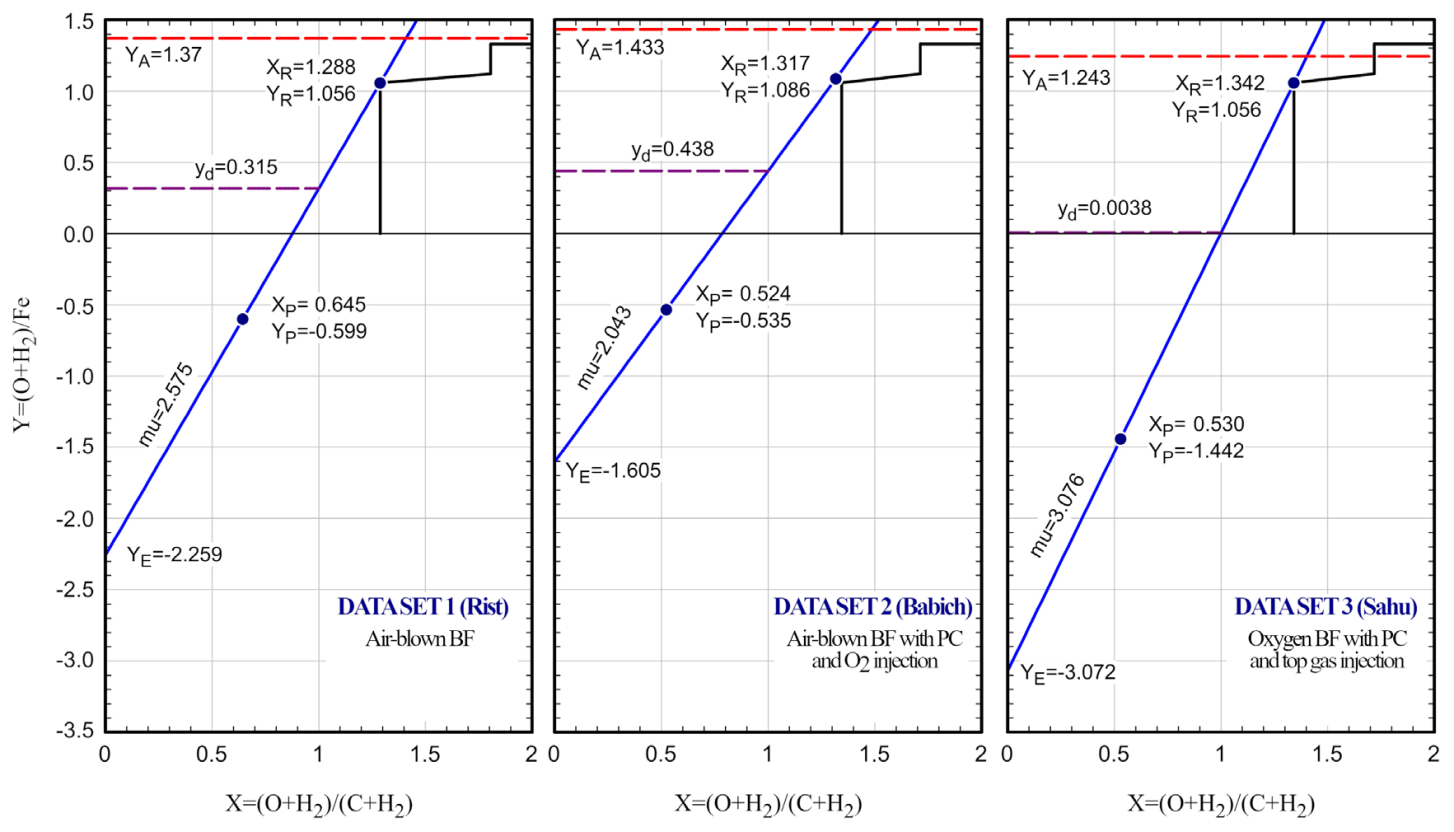
Operating lines obtained from the Data sets 1, 2 and 3 ( Table 3 and Table 4).

The operating line of this blast furnace is depicted in Figure 5, showing that 23% of the oxygen bonded to iron oxides is removed by direct reduction, and the remaining 77% through indirect reduction. The chemical efficiency was assumed 1, so the operating line passes through the point W of the equilibrium line.

### 5.2 Air-blown blast furnace with pulverized coal injection and O _2_-enriched air (Babich, Data set 2)

This data set was elaborated from the work of Babich *et al.* ^18^, corresponding to an air-blown blast furnace with pulverized coal injection and O _2_-enriched air. We consider the O _2_-enriched hot blast as an auxiliary injection. The proximate analysis of the coal is 74.1% Ω _FC_, 17.2% Ω _VM_, 7.6% Ω _Z_ and 1.2% Ω _M_^18^. Typical mass fractions for iron ore and coke were assumed. Moreover, since the operating parameters *r*, *T _R_* and *θ _st_* are not provided in the work of Babich *et al.*, typical values are chosen.

All results show a discrepancy below the 1% with respect to the data of Babich *et al.* ^18^, except for the coke consumption, which varies a 2.1%. The flame temperature calculated by Eq.(62) gives the same result than the one provided by Babich, since he developed that formula. The error in the flame temperature calculated by our model is 1%. The operating line of this blast furnace is depicted in Figure 5, which no longer passes through the point W of the equilibrium line (chemical efficiency is 0.92). The direct reduction represents a 30.6% and the indirect reduction a 69.4%.

### 5.3 Oxygen blast furnace with pulverized coal injection and top gas recycling (Sahu, Data set 3)

**Figure 6.**
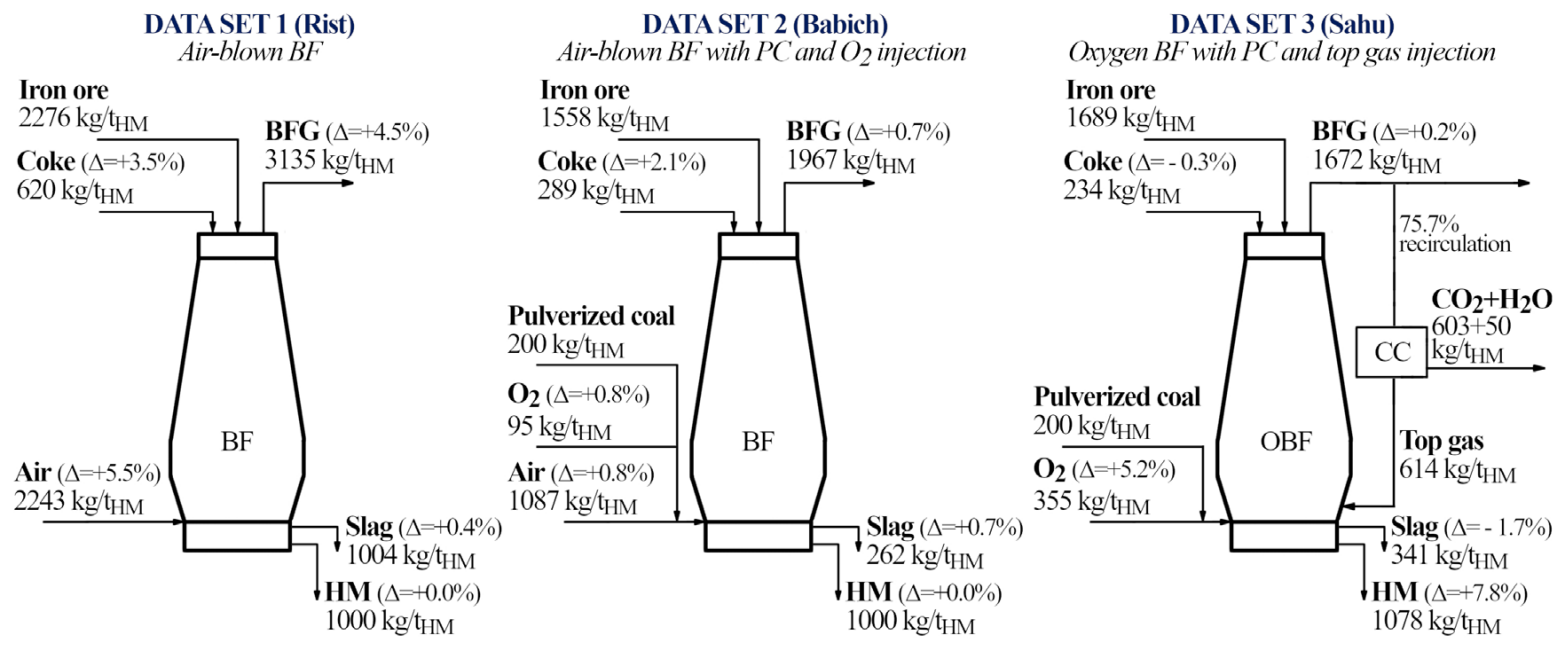
Conceptual schemes summarizing the mass flows and relative errors obtained during the validation of the model for Data set 1, 2 and 3 (the complete data sets are presented in Table 3 and Table 4).

In order to validate the model also under oxygen regimes, a third data set from the literature is used. The availability of complete data sets in literature is limited (probably because of non-disclosure agreements) and the information gets even more scarce when it comes to oxygen blast furnaces. For this reason, we take the only full data set we found (Sahu *et al.* ^27^), despite it has some underlying inconsistencies (which are consequently reflected in the results of our model). Nevertheless, it gives a good idea on the possibilities of the model when assessing oxygen blast furnaces. The data set corresponds to an oxygen blast furnace with pulverized coal injection and top gas recycling ( Figure 6). The proximate analysis of the coal is 58% Ω _FC_, 27% Ω _VM_, 10% Ω _Z_ and 5% Ω _M_.

The results of the model are compared to the reference data in Table 4. Most of the results have an error below the 8%, so it can be considered validated. The greatest discrepancy comes from the nitrogen content of the blast furnace (38% error) because the nitrogen mass balance is not correct in the data set provided by Sahu *et al.* ^26^. The total inlet mass of nitrogen is 17.6 kg/t _HM_ (from coal and top gas injection), while the total outlet mass was reported as 26.7 kg/t _HM_ (in the BFG). Assuming that the correct content of nitrogen in the BFG should be 17.6 kg/t _HM_, the relative error of the result of the model drops to a reasonable 6%. Other relevant inconsistencies are the incorrect mass balances of Fe, Mn and P. The former leads to an overestimation of the hot metal produced, and the latter to negative mass flows of MnO and P _2_O _5_ in the slag (the inlet mass flows of MnO and P _2_O _5_ are not enough to reach the specified Mn and P content in the hot metal). Despite these issues, the model can reproduce the behavior of the oxygen blast furnace described by Sahu *et al.* ^27^ within reasonable discrepancy limits. Moreover, the model has turn out to be a useful tool to identify potential inconsistencies in data sets.

**Table 4.** Data sets for oxygen blast furnaces with top gas recycling. The Data set 3 is taken from Sahu *et al*. ^27^ and compared with the results of our model. The Data sets 4, 5 and 6 were elaborated in the present paper.

	DATA SET 3			DATA SET 4	DATA SET 5	DATA SET 6
Input streams (kg/t _HM_)	Sahu ^27^	Model	Δ (%)	OBF-PtG	OBF-PtG with coke replacement	OBF-PtG with coal replacement
**Iron ore** (25°C)	**1689.4**	**1689.4**	**-**	**1500.0**	**1500.0**	**1500.0**
# Fe _2_O _3_	616.4	616.4	-	954.7	954.7	954.7
# FeO	771.8	771.8	-	369.2	369.2	369.2
# SiO _2_	78.8	78.8	-	58.9	58.9	58.9
# Al _2_O _3_	58.4	58.4	-	70.8	70.8	70.8
# CaO	129.4	129.4	-	15.7	15.7	15.7
# MgO	34.6	34.6	-	21.5	21.5	21.5
# MnO	-	-	-	9.2	9.2	9.2
**Coke** (25°C)	**234.9**	**234.1**	**-0.3**	**261.3**	**238.5**	**261.3**
# C	195.7	195.0	-0.3	217.7	198.7	217.7
# S	1.2	1.2	-0.3	1.3	1.2	1.3
# SiO _2_	22.8	22.7	-0.3	25.4	23.1	25.4
# Al _2_O _3_	13.6	13.6	-0.3	15.1	13.8	15.1
# CaO	1.1	1.1	-0.3	1.2	1.1	1.2
# MgO	0.5	0.5	-0.3	0.6	0.5	0.6
**Coal** (25°C)	**200.0**	**200.0**	**-**	**185.0**	**185.0**	**154.8**
# C	147.3	147.3	-	142.1	142.1	118.9
# H	9.0	9.0	-	5.9	5.9	4.9
# O	8.9	8.9	-	9.4	9.4	7.9
# N	4.0	4.0	-	4.7	4.7	3.9
# S	1.1	1.1	-	0.8	0.8	0.7
# H _2_O	10.0	10.0	-	2.2	2.2	1.9
# FeO	0.7	0.7		-	-	-
# SiO _2_	11.9	11.9	-	11.4	11.4	9.5
# Al _2_O _3_	6.2	6.2	-	8.2	8.2	6.9
# CaO	0.5	0.5	-	0.3	0.3	0.3
# MgO	0.3	0.3	-	-	-	-
# P _2_O _5_	0.1	0.1	-	-	-	-
**O _2_ injection** (25°C Sahu / 1200°C Bailera)	**337.6**	**355.3**	**5.2**	**334.7**	**345.4**	**340.0**
# O _2_	330.7	348.1	5.3	318.1	328.2	323.0
# H _2_O	6.9	7.3	4.6	7.7	8.0	7.8
# N _2_	-	-	-	9.0	9.3	9.1
**Recycled gas injection** (900°C Sahu / 1200°C Bailera)	**613.6**	**613.6**	**0.0**	**600.0**	**600.0**	**600.0**
# N _2_	13.6	12.5	-8.0	27.0	24.1	24.6
# CO _2_	34.2	31.7	-7.1	53.1	45.0	48.7
# CO	554.4	558.2	0.7	512.5	524.7	518.7
# H _2_	11.5	11.2	-2.5	7.5	6.3	8.0
**Synthetic methane injection** (25°C Bailera)	**-**	**-**	**-**	**-**	**21.8**	**21.8**
# CH _4_	-	-	-	-	21.1	21.1
# CO _2_	-	-	-	-	0.5	0.5
# H _2_O	-	-	-	-	0.1	0.1
# H _2_	-	-	-	-	0.1	0.1
Output (kg/t _HM_)						
**Hot metal** (1425°C Sahu / 1500°C Bailera)	**1000.0**	**1078.1**	**7.8**	**1002.3**	**1002.3**	**1002.3**
# Fe	949.5	1023.7	7.8	951.2	951.2	951.2
# C	42.3	45.6	7.9	44.7	44.7	44.7
# Si	5.8	6.3	7.9	5.8	5.8	5.8
# Mn	0.6	0.7	8.3	0.6	0.6	0.6
# P	1.8	1.9	0.0	-	-	-
**Slag** (1500°C Sahu / 1550°C Bailera)	**347.8**	**341.3**	**-1.9**	**226.1**	**222.4**	**222.8**
# FeO	-	0.7	-	-		
# SiO _2_	-	100.1	-	83.2	81.0	81.4
# Al _2_O _3_	-	78.1	-	94.1	92.8	92.8
# CaO	-	127.0	-	13.5	13.5	13.6
# MgO	-	35.4	-	22.1	22.0	22.1
# MnO	-	-0.8	-	8.4	8.4	8.4
# CaS	-	5.2	-	4.8	4.5	4.5
# P _2_O _5_	-	-4.3	-	-		
**BFG** (100°C Sahu / 150°C Bailera)	**1668.1**	**1672.0**	**0.2**	**1652.7**	**1666.0**	**1652.7**
# N _2_	26.7	16.5	-38.2	40.6	38.0	37.6
# CO _2_	798.7	838.1	4.9	799.8	710.7	745.0
# CO	762.2	737.0	-3.3	772.1	828.6	793.4
# H _2_O	66.7	65.7	-1.5	28.9	78.8	64.5
# H _2_	13.8	14.7	7.0	11.3	9.9	12.2
Operating conditions						
Chemical efficiency, *r* (-)	-	1.000	-	0.920	0.920	0.920
Thermal reserve zone temperature, T _R_ (°C)	807.6	807.6	-	950.0	950.0	950.0
Flame temperature, T _fl_ (°C)	2126.0	1768.9	-16.8	2082.9	1995.6	2014.2
Heat evacuated by the staves (MJ/THM)	500.0	500.0	-	700.0	700.0	700.0
Heat evacuated in the elaboration zone, *θ* *_st_* (-)	-	0.770	-	0.800	0.800	0.800
Oxidation state of the BFG, *η*_CO–H _2__ (-)	0.391	0.403	3.2	0.374	0.373	0.374

Regarding the flame temperature, in this case we cannot use the Eq.(62) to compute it since this formula does not account for top gas injections. By using our model, we found a flame temperature of about 1770°C, which is a 17% lower than the one reported by Sahu *et al.* ^27^ (2126°C). Although they did not explain how they calculated this temperature, they probably did not account for the heating of the injected top gas, while our model does. In fact, one of the reasons of recirculating top gas is to substitute N _2_ as heat sink during oxygen regimes, so probably that is why Sahu *et al.* ^26^ had to set the inlet temperature of the oxygen to 25°C (otherwise they would found excessive flame temperatures because of not accounting for the recycled gas). If we calculate in our model the temperature of the flame without taking into account the sensible heat of the recycled gas, we found temperatures between 2200 and 2300°C, which are closer to that provided by Sahu *et al.* ^26^.

The operating line of this blast furnace is depicted in Figure 5. It passes through the point W of the equilibrium line because it is assumed chemical ideality. It can be seen that that the direct reduction represents only a 0.3% of the total reduction, which is unrealistic for a real operation. Therefore, for this reason and the other mentioned inconsistencies, we do not recommend using this data set for further analyses.

## 6. Predicting the operating line for an oxygen blast furnace integrated with PtG

Once the model has been validated, we are going to analyze a potential configuration for a blast furnace operation under an oxygen regime, with top gas recycling, and injections of pulverized coal and synthetic natural gas. The data set was inspired in the works of Sahu *et al.* ^27^ and Jin *et al.* ^29^, aiming to reproduce similar results to them, but keeping reasonable operating conditions ( *e.g*., flame temperature between 2000–2300°C, direct reduction about 10–15%, slag production above 200 kg/t _HM_). The proximate analysis of the coal for this data set is 70.7% Ω _FC_, 17.2% Ω _VM_, 10.8% Ω _Z_ and 1.2% Ω _M_.

**Figure 7.**
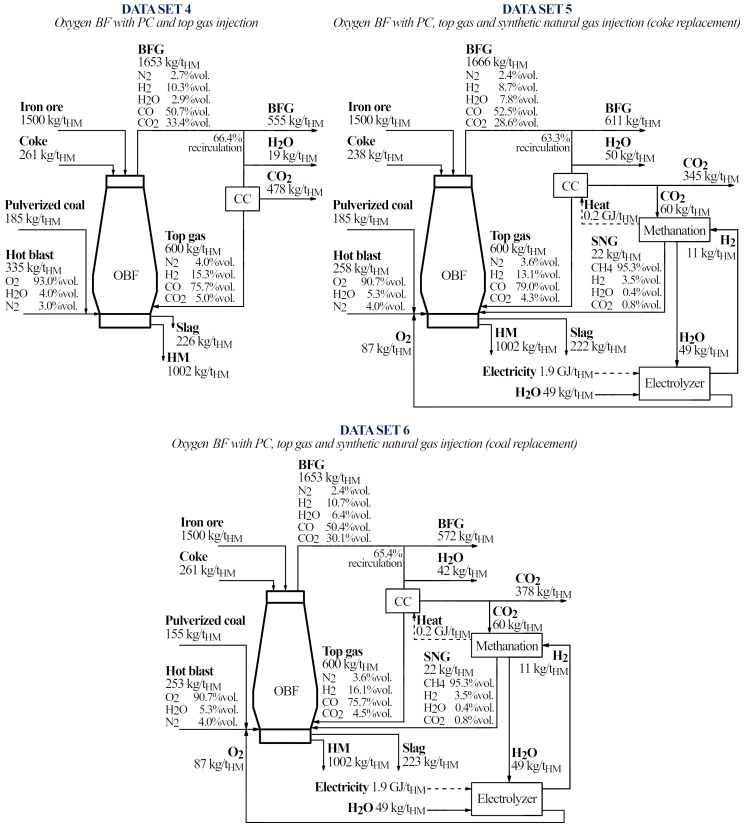
Conceptual schemes summarizing the mass flows obtained for Data sets 4, 5 and 6 (see Table 4).

The proposed oxygen blast furnace would be integrated with a PtG plant, which renewably produces the synthetic methane. This methane is used to reduce the consumption of coke or coal, thus substituting a fossil fuel by a renewable fuel. The conceptual scheme of the blast furnace is the one depicted in Figure 7 (Data set 5 when coke is replaced and Data set 6 when coal is replaced). We assume a 150 MW electrolyzer and a 280 t _HM_/h blast furnace. This means that the electrolyzer produces 11 kg/t _HM_ of hydrogen, which are converted to 21.8 kg/t _HM_ of synthetic methane by consuming 60 kg/t _HM_ of CO _2_. This carbon is continuously recycled in a closed loop, and therefore the corresponding emissions are avoided. The CO _2_ would come from the carbon capture stage that is used to recycle the top gas, which can benefit from the exothermal heat available from the methanation process. Furthermore, the electrolyzer by-produces 87 kg/t _HM_ of O _2_ that can be used in the blast furnace, thus reducing the energy requirements in the air separation unit that enriches the hot blast. For these calculations, it was assumed an electrolysis efficiency of 68% ^30^.

For comparison purposes, the blast furnace was also modelled without the integration of the PtG plant, and therefore without the injection of synthetic methane (Data set 4, which is considered as the base case). In all cases, the mass flow of injected top gas was kept constant at 600 kg/t _HM_ (despite its composition changes). The three data sets are presented in Table 4. In the base case (Data set 4), the total CO _2_ that would be emitted after the combustion of the BFG is 1154 kg _CO2_/t _HM_, of which the 41.4% ( *i.e*., 478 kg _CO2_/t _HM_) could be directly captured since they come from the carbon capture stage. When implementing PtG, 60 kg/t _HM_ of CO _2_ are recycled in closed loop, which allow to reduce the coke consumption by 8.7% or alternatively the coal consumption by 16.3%. In case of substituting coke, the total emissions become 1083 kg _CO2_/t _HM_, of which the 31.8% can be directly captured and stored ( *i.e.*, 345 kg _CO2_/t _HM_). Thus, in overall terms, the CO _2_ is diminished by 71 kg _CO2_/t _HM_ with respect to the base case by using 1.93 GJ/t _HM_ of electricity in the electrolyzer, so the energy penalty of the CO _2_ avoidance is 27.1 MJ/kg _CO2_. This value is line with the results of Perpiñán *et al.* ^8^ for similar PtG integrations in the steel industry (he reported 34 MJ/kg _CO2_). Actually, since we have cut the coke consumption by 22.8 kg/t _HM_, which, in terms of electricity, is equivalent to a reduction of 0.24 GJ/t _HM_ (assuming a coke heating value of 27.3 MJ/kg and a subcritical power plant net efficiency of 38% ^31^), and additionally we diminished the O _2_ that has to be produced in the air separation unit by 77 kg _O2_/t _HM_ (we need inject 10 kg _O2_/t _HM_ more than in the base but we have 87 kg _O2_/t _HM_ available from electrolysis), which translates into a reduction of 0.05 MJ/t _HM_ of electric consumption (assuming a typical ASU specific consumption of 0.61 MJ/kg _O2_^32^), the net energy penalization of the CO _2_ avoidance becomes 23.1 MJ/kg _CO2_. Alternatively, in the case of using the synthetic methane to replace part of the coal, the total emissions become 1068 kg _CO2_/t _HM_. Following the same calculations just mentioned, the gross energy penalization of CO _2_ avoidance in this case would be 22.4 MJ/kg _CO2_ and the net energy penalization 17.9 MJ/kg _CO2_ (the heating value of coal is 29.4 MJ/kg, Eq.(130)). Table 5 summarizes these calculations.

**Table 5.** Comparison of CO _2_ emissions, CO _2_ avoided and energy penalization between data sets 4 (oxygen blast furnace), 5 (oxygen blast furnace with PtG integration for the replacement of coke by synthetic methane) and 6 (oxygen blast furnace with PtG integration for the replacement of coal by synthetic methane).

	OBF Base case (Data set 4)	OBF-PtG with coke replacement (Data set 5)	OBF-PtG with coal replacement (Data set 6)
Total CO _2_ emissions (kg/t _HM_)	1154	1083	1068
- From BFG combustion (kg/t _HM_)	676	738	690
- From CC stage (kg/t _HM_)	478	345	378
Total CO _2_ emissions avoided (kg/t _HM_)	-	71	86
Total CO _2_ emissions avoidance ratio (%)	-	6.1	7.4
Electricity for PtG (GJ/t _HM_)	-	1.93	1.93
Electricity saved in the ASU (GJ/t _HM_)	-	0.05	0.05
Fossil fuel saved (kg/t _HM_)	-	22.8	30.2
- Equivalent electricity saved (GJ/t _HM_)	-	0.24	0.34
Fossil fuel replacement ratio (kg/kg _SNG_)	-	1.05	1.38
Gross CO _2_ avoidance penalization (MJ/kg _CO2_)	-	27.1	22.4
Net CO _2_ avoidance penalization (MJ/kg _CO2_)	-	23.1	17.9

These values of energy penalization for the avoidance of CO _2_ are clearly above the typical consumptions of conventional amine carbon capture, which are usually in the range 3.7–4.8 MJ/kg _CO2_^33, 34^. Nevertheless, the integration of PtG presents the additional benefits of diminishing the coke/coal consumption in the blast furnace, reducing the electricity consumption in the air separation unit, and suppressing the requirement of geological storage for the avoided CO _2_ (it is kept in a closed carbon loop thanks to PtG). Therefore, an overall economic and energy analysis of the whole integrated steel plant should be necessary to reach farther conclusions. Furthermore, there exist other potential PtG integrations that may lead to lower energy penalizations, such as the utilization of the BFG in the methanation stage rather than the captured CO _2_ (which allow producing more SNG with the same amount of H _2_) or the injection of the H _2_ in the blast furnace without considering a methanation stage.

Regarding the flame temperature, it is reduced about 90°C when injecting 22 kg/t _HM_ of synthetic methane for replacing coke. This is a reduction of 4.0°C per kg _SNG_/t _HM_, which is in good agreement with the value reported by Babich *et al.* for natural gas (4.5°C per kg _NG_/t _HM_^16^). When we replace coal by synthetic natural gas, the temperature of the flame is reduced by 3.1°C per kg _SNG_/t _HM_, which also matches the value reported by Babich *et al.* for this type of replacement (3.0°C per kg _NG_/t _HM_^16^).

**Figure 8.**
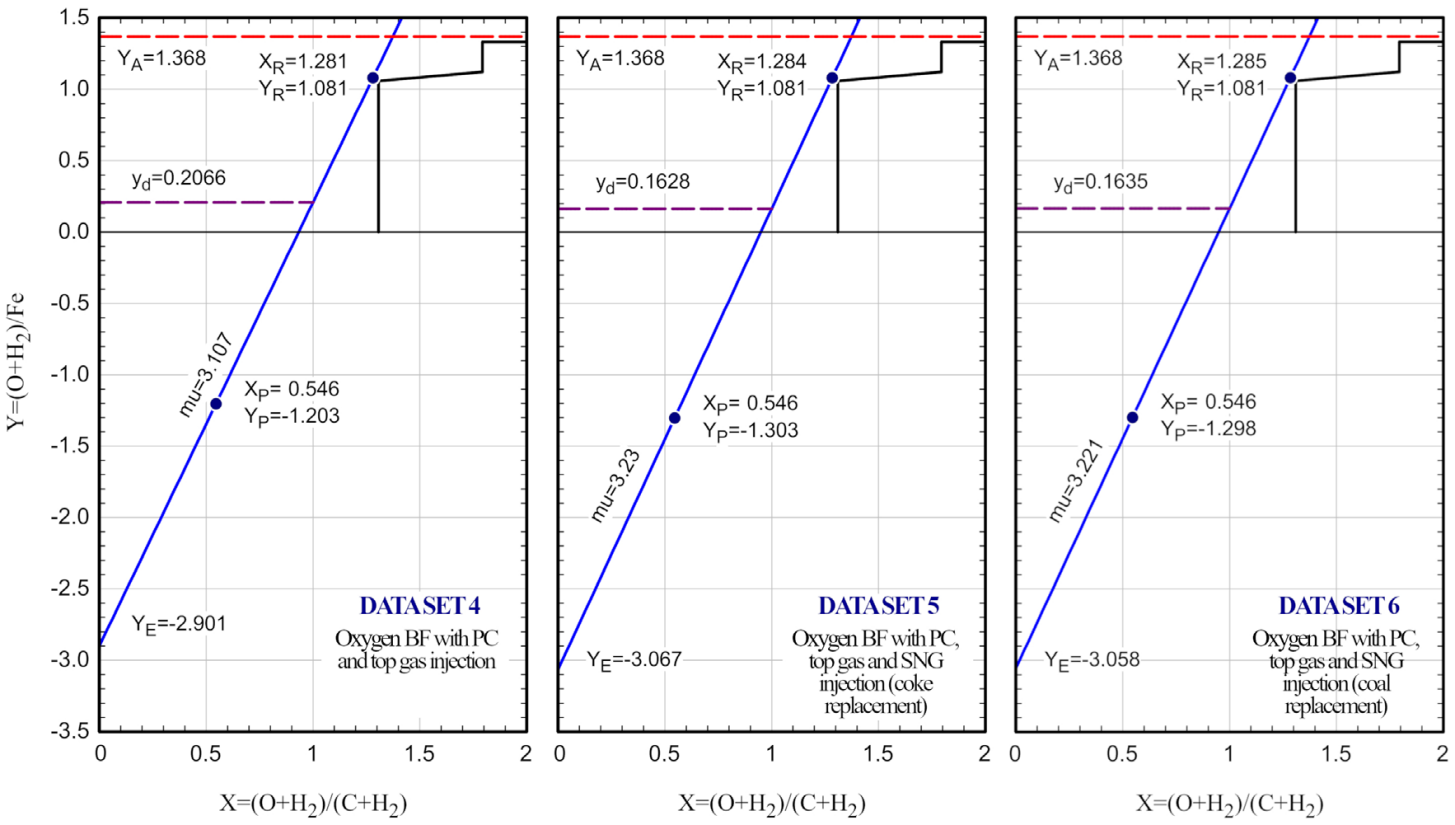
Operating lines of oxygen blast furnaces, obtained from Data sets 4, 5 and 6 ( Table 4).

Lastly, the percentage of direct reduction is 15% for the base case and about 12% when injecting synthetic natural gas. The decrease in this value was expected since direct reduction takes place through solid carbon, and the coke/coal input is diminished when PtG is integrated. The operating lines are presented in Figure 8.

## 7. Conclusions

The operating diagram, which was originally developed by Rist in 1963, is a useful methodology for predicting the operating conditions in a blast furnace through a graphical representation which takes into account the mass and energy balances. This methodology was described in a series of papers between 1963 and 1967, of which the most relevant were published in French. In this paper we have revisited the work of Rist, summarizing his methodology and making some additions and corrections. We have presented a general model that considers multiple injectants separately, with all calculations given as a function of the temperature of the thermal reserve zone. Besides, the model now calculates the sensible heats of the hot metal and slag as a function of their composition, and the heat of carburization as a function of the austenite and cementite content in iron. Furthermore, it is added a supplementary model to compute the heat of decomposition of coal, an additional energy balance in the upper zone of the blast furnace to compute the final composition of the blast furnace gas, and other energy balance for the calculation of the flame temperature. Regarding corrections, the heat associated to the direct reduction of FeO now accounts for the moisture of the hot blast, the heat associated to the lack of chemical ideality now includes the influence of the hydrogen coming from auxiliary fuels and of the moisture of the hot blast, and lastly, the sensible heats of hot metal and slag are now correctly computed and accounted.

The model elaborated in this paper has been validated with three different operation data sets from literature. The first one corresponds to an air-blown blast furnace without auxiliary injections (error <5.6%), the second one to an air-blown blast furnace with pulverized coal injection (error <2.1%), and the third one to an oxygen blast furnace with top gas recycling and pulverized coal injection (error <8.3%). However, it would be desirable to have a discrepancy below 5% in all cases, we consider the model validated since the slightly variation above 5% is justified. In the first case, the data set corresponds to the results of Rist, therefore they include the errors that were corrected in our model. When we deliberately include in our model the same errors than Rist, the difference between both results becomes lower than 3.5%. In the third case, the reference data set have some incorrect mass balances in origin that led to this higher error. Nevertheless, these inconsistencies were properly identified by our model, and the overall behavior of the blast furnace could be correctly described.

The objective in revisiting the operating diagram of Rist is to study a new concept for the reduction of CO _2_ emissions in blast furnaces. This concept combines oxygen blast furnaces with PtG technology. The latter renewably produces synthetic methane, which is used to replace part of the coke or coal. Carbon is thus continuously recycled in a closed loop, and the corresponding emissions are avoided without requiring geological storage. Furthermore, the electrolyzer of the PtG plant by-produces O _2_ that can be used in the blast furnace to reduce the electricity consumption in the air separation unit that enriches the hot blast. In this study, we presented the first approach to this integration concept, comparing the performance of the blast furnace when coke is replaced to the performance when coal is replaced. It was assumed a 280 t _HM_/h oxygen blast furnace producing 1154 kg _CO2_/t _HM_, coupled to a PtG plant with 150 MW electrolysis power capacity that produces 11 kg _H2_/t _HM_ of hydrogen (then converted to 21.8 kg _SNG_/t _HM_ of synthetic methane by using pure CO _2_ from a carbon capture stage). The model shows fuel replacement ratios of 1.05 kg/kg _SNG_ for coke and 1.38 kg/kg _SNG_ for coal, which lead to CO _2_ emission reductions of 6.1% and 7.4%, respectively. As the electricity consumed in the PtG plant is 1.93 GJ/t _HM_, the gross energy penalization of the CO _2_ avoidance is 27.1 MJ/kg _CO2_ when coke is replaced and 22.4 MJ/kg _CO2_ when coal is replaced. Considering the energy content of the fossil fuel that is saved, and the electricity that is no longer consumed in the air separation unit, the net energy penalizations are 23.1 MJ/kg _CO2_ and 17.9 MJ/kg _CO2_, respectively. These values are several times greater than the specific consumption of amine carbon capture (typically 3.7–4.8 MJ/kg _CO2_). However, the integration of PtG diminishes the coke/coal consumption in the blast furnace, reduces the electricity consumption in the air separation unit, and eliminates the requirement of geological storage for the avoided CO _2_. Therefore, a detailed economic comparison between both methods should be necessary to reach firm conclusions. Furthermore, there exist other potential PtG integrations that may lead to lower energy penalizations, such as the utilization of the BFG in the methanation stage rather than captured CO _2_, what would allow producing more SNG with the same amount of H _2_.

Lastly, the present paper has provided six full data sets of different blast furnaces operations, specifying the composition of all streams, as well as the most relevant operating parameters ( *e.g.*, temperature of the thermal reserve zone, heat evacuated by the staves, and the temperature of the flame). The availability of this information in literature is very scarce, especially for oxygen blast furnaces.

## Nomenclature

In this paper we used calorie as unit of energy for calculations related to operating diagram in order to facilitate comparing results with the original work of Rist. Elsewhere, the SI unit is used (Joule).

### Symbols

*a _i_*             calculation parameter, 1/°C ^i^

a _j_             number of moles of H _2_ in injectant j per number of moles of injectant j, mol _H2_/mol _j_

A             calculation parameter, kcal/mol

b _j_             number of moles of O _2_ in injectant j per number of moles of injectant j, mol _O2_/mol _j_

B             calculation parameter, kcal/mol

c _j_             number of moles of N _2_ in injectant j per number of moles of injectant j, mol _N2_/mol _j_

*c _p_*             heat capacity, kcal/(mol·K)

C              calculation parameter, kcal/mol

*C*_Δ *T _R_*_        sensible heat of the burden between *T _R_* – Δ *T _R_* and *T _R_* (lack of thermal ideality), kcal/mol _Fe_

d _j_              number of moles of S _2_ in injectant j per number of moles of injectant j, mol _S2_/mol _j_

*e*               number of moles of H _2_O per number of moles of O in the air (i.e., *e* = *y _e_*/ *y _v_*), mol _H2O_/mol _O_

*f*                sensible heat of the hot metal between *T _R_* and *T _f_* (outlet temperature), kcal/mol _Fe_

*g _i_*              specific Gibbs free energy of compound i, kcal/mol _i_

Δ *G*            Gibbs free energy change of reaction, kcal/mol 

*h*               enthalpy, kcal/mol 

Δ *h _f_*            enthalpy of fusion, kcal/mol 

Δ _c_ *h*           enthalpy of combustion, kcal/mol

Δ _f_ *h*            enthalpy of formation, kcal/mol

*K*_eq_            equilibrium constant of the water-gas shift reaction, -

*l*                sensible heat of the slag between *T _R_* and *T _l_* (outlet temperature), kcal/mol _Fe_

*m _j,i_*            mass of compound *i* in stream *j* per ton of hot metal, kg/t _HM_

*M*              molar weight, kg/kmol

*n _j,i_*             number of moles of compound *i* in stream *j* per ton of hot metal, mol/t _HM_

*p*                heat removed by the staves in the elaboration zone, kcal/mol _Fe_

$p^{\prime }$ heat removed by the staves in the preparation zone, kcal/mol _Fe_

*q _c_*               heat released at *T _R_* by the reaction C (coke) + 0.5 O _2_ → CO, kcal/mol _O_

*q _e_*               heat required by the H _2_O in hot blast due to dissociation, reverse water-gas shift and sensible heat, kcal/mol _H2O_

*q _er_*              heat absorbed at *T _R_* by the reaction C + H _2_O → CO + H _2_, kcal/mol _H2O_

*q _es_*              enthalpy change of water between *T _v_* and *T _R_*, kcal/mol _H2O_

*q*_*f*,i_              heat required to melt and increase the temperature of compound *i* from *T _R_* to *T _f_*, kcal/mol _i_

*q*_Fe _0.95_O_    sensible heat of wüstite between *T _R_* – Δ *T _R_* and *T _R_*, kcal/mol _O_

*q*_Fe _3_C_         heat absorbed at *T _R_* by the reaction 3Fe + C → Fe _3_C, kcal/mol _C_

*q*_Fe _3_O _4__       sensible heat of magnetite between *T _R_* – Δ *T _R_* and *T _R_*, kcal/mol _Fe3O4_

*q _g_*                heat absorbed at *T _R_* by the reaction C + CO _2_ → 2CO, kcal/mol _C_

*q _im_*               heat released at *T _R_* by the reaction 1/4 Fe _3_O _4_ + CO → 3/4 Fe + CO _2_, kcal/mol _O_

*q _iw_*               heat released at *T _R_* by the reaction Fe _0.95_O + CO → 0.95 Fe + CO _2_, kcal/mol _O_

*q _j_*                 thermal demand by injectant *j*, CH _2a_O _2b_N _2c_S _2d_Z _z_ (or H _2a_O _2b_N _2c_ or O _2b_ N _2c_) due to dissociation, sensible heat, reverse water-gas shift of the H _2_, incomplete combustion with the O _2_, and transfer of S to the slag, kcal/mol _j_

*q _jd_*               heat absorbed at *T _j_* by the dissociation reaction of injectant *j*, CH _2a_O _2b_N _2c_S _2d_Z _z_ → C + aH _2_ + bO _2_ + cN _2_ + dS _2_ + zZ, kcal/mol _j_

*q _js,i_*              enthalpy change of element *i* from injectant *j* between *T _j_* and *T _R_*, kcal/mol _i_

*q _k_*                 heat absorbed at *T _R_* by the total H _2_ in the furnace when considering that it is completely converted to H _2_O through the reverse water-gas shift reaction, kcal/mol _H2_

*q*_*l*,i_                heat required to melt and increase the temperature of compound *i* (SiO _2_, Al _2_O _3_, CaO, MgO) from *T _R_* to *T _l_*, kcal/mol _i_

*q*_Mn_              heat absorbed at *T _R_* by the reaction C +MnO → CO + Mn, kcal/mol _O_

*q _P_*                 heat absorbed at *T _R_* by the reaction 1/5 P _2_O _5_ ⋅ 3CaO +C + 6/5 Fe → 2/5 Fe _3_P + 3/5 CaO + CO, kcal/mol _O_

*q _rmc_*              heat consumed by the reaction 1.5 Fe _2_O _3_ + 0.5 CO → Fe _3_O _4_ + 0.5 CO _2_, including also the heat exchange between reactants and products at different temperatures, kcal/mol _Fe3O4_

*q _rwc_*              heat consumed by the reaction 0.5 Fe _2_O _3_ + 0.5 CO → FeO + 0.5 CO _2_, including also the heat exchange between reactants and products at different temperatures, kcal/mol _FeO_

*q*_*R*,i_               sensible heat of compound *i* (SiO _2_, Al _2_O _3_, CaO, MgO, C) between *T _R_* – Δ *T _R_* and *T _R_*, kcal/mol _i_

*q _S_*                 heat absorbed at *T _R_* by the reaction 1/2 S _2_ + CaO + C → CaS + CO, kcal/mol _S_

*q _s,i_*               sensible heat of component i between *T*_fl_ and *T _R_*, kcal/mol _i_

*q _s,nr,i_*            sensible heat of non-reacting compound i, kcal/mol _i_

*q*_Si_                heat absorbed at *T _R_* by the reaction C + 1/2 SiO _2_ + 3/2 Fe → CO + 1/2 Fe _3_Si, kcal/mol _O_

*q _st_*                 heat removed by the staves from the blast furnace per ton of hot metal, kcal/t _hot metal_

*q _v_*                  energy available from the sensible heat of the air between *T _v_* and *T _R_*, kcal/mol _O_

*q _y_*                  heat absorbed at *T _R_* by the carburization of the iron, kcal/mol _C_

*q* *_y_* Fe              heat absorbed at *T _R_* by the reaction C(coke) → C(austenite), kcal/mol _C_

*q _ε_*                  heat absorbed at *T _R_* by the reaction H _2_ + CO _2_ → H _2_O + CO, kcal/mol _H2_

${q^{\prime }}_{\varepsilon }$ heat absorbed by the reaction H _2_ + CO _2_ → H _2_O + CO taking into account the temperature of reactants and products, kcal/mol _H2(reacting)_

*r*                    chemical efficiency of the blast furnace, -

*R*                   ideal gas constant, kcal/(mol K)

*S _i_*                  specific entropy of compound i, kcal/(mol _i_ K)

*T*                   temperature, °C (unless otherwise specified)

*ΔT _R_*              difference of temperature between the gas and the solid at the beginning of the middle zone, °C

*V _j_*                  volume flow of stream *j* consumed in the blast furnace per ton of hot metal, Nm ^3^/t _HM_

*x _d_*                  number of moles of O removed from wüstite by direct reduction per total moles of reducing gas mixture, mol _O_/(mol _C_+mol _H2_)

*x _e_*                  number of moles of H _2_O in hot blast per total moles of reducing gas mixture, mol _H2O_/(mol _C_+mol _H2_)

*x _h_*                  molar fraction of hydrogen and water in the reducing gas mixture, -

*x _i_*                   number of moles of O transferred from the iron oxides to the gas by indirect reduction per total moles of reducing gas mixture, mol _O_/(mol _C_+mol _H2_)

*x _j_*                   number of moles of injectant j (overall formula CH _2a_O _2b_N _2c_S _2d_Z _z_, H _2a_O _2b_N _2c_ or O _2b_N _2c_) per total moles of reducing gas mixture, mol _j_/(mol _C_+mol _H2_)

*x _k_*                  number of moles of H _2_ in the coke per total moles of reducing gas mixture, mol _H2_/(mol _C_+mol _H2_)

*x _Mn_*                number of moles of O removed by direct reduction of MnO per total moles of reducing gas mixture, mol _O_/(mol _C_+mol _H2_)

*x _P_*                  number of moles of O removed by direct reduction of P _2_O _5_ per total moles of reducing gas mixture, mol _O_/(mol _C_+mol _H2_)

*x _S_*                  number of moles of O replaced by S in the slag per total moles of reducing gas mixture, mol _O_/(mol _C_+mol _H2_)

*x _Si_*                 number of moles of O removed by direct reduction of SiO _2_ per total moles of reducing gas mixture, mol _O_/(mol _C_+mol _H2_)

*x _v_*                  number of moles of O in hot blast per total moles of reducing gas mixture, mol _O_/(mol _C_+mol _H2_)

X                   abscissa in the Rist diagram, (mol _O_+mol _H2_)/(mol _C_+mol _H2_)

*y*_C_                 number of moles of C (coke) that are consumed in combustion in the raceways per mol of Fe produced, mol _C_/mol _Fe_

*y _d_*                  number of moles of O removed from wüstite by direct reduction per mol of Fe produced, mol _O_/mol _Fe_

*y _e_*                  number of moles of H _2_O in hot blast per mol of Fe produced, mol _H2O_/mol _Fe_

*y _i_*                   number of moles of O transferred from the iron oxides to the gas by indirect reduction per mol of Fe produced, mol _O_/mol _Fe_

*y _j_*                   number of moles of injectant j (overall formula CH _2a_O _2b_N _2c_S _2d_Z _z_, H _2a_O _2b_N _2c_ or O _2b_N _2c_) per mol of Fe produced, mol _j_/mol _Fe_

*y _k_*                   number of moles of H _2_ in the coke per mol of Fe produced, mol _H2_/mol _Fe_

*y _Mn_*                number of moles of O removed by direct reduction of MnO per mol of Fe produced, mol _O_/mol _Fe_

*y _nr,i_*                number of moles of compound i that is traversing the preparation zone without reacting per mol of Fe produced, mol _i_/mol _Fe_

*y _P_*                  number of moles of O removed by direct reduction of P _2_O _5_ per mol of Fe produced, mol _O_/mol _Fe_

*y*_rg,i_                number of moles of component *i* in the reducing gas at the raceways per mol of Fe produced, mol _i_/mol _Fe_

*y _rmc_*               number of moles of Fe _3_O _4_ produced in the preparation zone per mol of Fe produced, mol _Fe3O4_/mol _Fe_

*y _rwc_*                number of moles of FeO produced in the preparation zone per mol of Fe produced, mol _FeO_/mol _Fe_

*y _S_*                   number of moles of O replaced by S in the slag per mol of Fe produced, mol _O_/mol _Fe_

*y _Si_*                  number of moles of O removed by direct reduction of SiO _2_ per mol of Fe produced, mol _O_/mol _Fe_

*y _v_*                   number of moles of O in hot blast per mol of Fe produced, mol _O_/mol _Fe_

*y _wgs_*               number of moles of H _2_ reacting in the preparation zone, mol _H2_/mol _Fe_

Y                   ordinate in the Rist diagram, (mol _O_+mol _H2_)/mol _Fe_

Y _E_                 intercept of the operating line representing the moles of H _2_ and O coming from sources other than iron oxides that contribute to the formation of the reducing gas per mol of Fe produced (negative sign by convention), (mol _O_+mol _H2_)/mol _Fe_

$Y_{\text{E}}^{*}$ terms of Y _E_ that are independent of *y _v_*, (mol _O_+mol _H2_)/mol _Fe_

$Y_{M}^{*}$ number of moles of O per mole of Fe _3_O _4_ (i.e., $Y_{M}^{*}$ = 4), mol _O_/mol _Fe3O4_

Z _j_                  number of moles of ashes in injectant j per number of moles of injectant j, mol _ash_/mol _j_

### Greek symbols

*γ*                 number of moles of C dissolved in the hot metal, mol _C_/mol _Fe_

*γ*_Fe _3_C_          number of moles of C dissolved in the hot metal as cementite, mol _C_/mol _Fe_

*γ*_*γ*Fe_             number of moles of C dissolved in the hot metal as austenite, mol _C_/mol _Fe_

*δ*                 decrease in the available heat due to the presence of magnetite in the elaboration zone, kcal/mol _Fe_

$\delta^{\prime }$ decrease in the heat absorbed by the reverse water-gas shift reaction because of the lack of chemical ideality, kcal/mol _Fe_

Δ _1_               calculation parameter, -

Δ _2_               calculation parameter, (mol _O_+mol _H2_)/mol _Fe_

_K_               number of moles of C and H _2_ in coke per kg of coke, (mol _C_+mol _H2_)/mol _K_

*η*                  humidity in the air, (g/Nm ^3^)

*η*_CO–H _2__       oxidation state of the blast furnace gas leaving the top, -

*θ _st_*               fraction of the total heat removed by the staves that is coming from the elaboration zone (i.e., from the control volume of the energy balance), -

μ                 slope of the Rist diagram, *i.e.* , number of moles of reducing gas per mol of Fe produced, (mol _C_+mol _H2_)/mol _Fe_

*τ _j_*                 calculation parameter that is 1 when the auxiliary injection *j* contains carbon and 0 when not, -

*ϕ*                 percentage of H _2_ consumed in the preparation zone, -

*ω*                molar fraction, -

Ω _ji_              mass fraction of compound *i* in stream *j*, -

### Subscripts and superscripts

A          initial oxidation state of the iron oxides at the inlet of the blast furnace

BFG     blast furnace gas

*C*           related to carbon or C-CO _2_ mixtures

*d*           dry basis (superscript), decomposition (subscript)

*e*           moisture in hot blast

*f*            hot metal (at the outlet)

*FC*        fixed carbon

Fe _3_C     referred to the number of moles of C in hot metal dissolved as cementite

fl           flame

*H*           related to hydrogen or H _2_-H _2_O mixtures

HM       hot metal

IN          inlet of solids at the top of the furnace

IO          iron ore

*j*             injectant (overall formula CH _2a _j__ O _2b _j__ N _2c _j__ S _2d _j__ Z _Z _J__ or stream

K           coke

slag       (at the outlet)

*l*             magnetite or moisture

*m*           natural moisture in blast

NG        natural gas

*nr*          non-reacting in the preparation zone

*P*           characteristic point of the operating line referring to the energy balance of the blast furnace

*PM*        primary volatile matter

*R*           characteristic point of the operating line referring to the thermal reserve zone

*SM*        secondary volatile matter

*v*            hot blast / air

*VM*        volatile matter

*W*           referring to wüstite or to the characteristic point of the equilibrium line in which pure wüstite is in equilibrium with the gas

*P*            ashes

*P*γFe       referred to the number of moles of C in hot metal that are dissolved as austenite

## Data availability

### Extended data

Zenodo: Extended data for ‘Revisiting the Rist diagram for predicting operating conditions in blast furnaces with multiple injections’ https://doi.org/10.5281/zenodo.5637580 ^39^

The project contains the following extended data:

      Appendix A – Terms of the energy balance in the elaboration zone

      Appendix B – Heat of decomposition of coal (dry basis)

      Appendix C – Energy balance in the preparation zone

      Appendix D – Calculation of the flame temperature through Eq.(62)

      DISIPO_OPEN DATA_1.xlsx

Data are available under the terms of the Creative Commons Attribution 4.0 International license (CC-BY 4.0).
