# Supplementary material for: Revisiting the Rist diagram for predicting operating conditions in blast furnaces with multiple injections
Source: Open Res Eur. 2021 Nov 29;1:141. [Version 1] doi: 10.12688/openreseurope.14275.1 (PMC10445826; doi:10.12688/openreseurope.14275.1)
Supplement: Peer review history

# Peer review: Reviewer response for version 1

**DOI:** 10.21956/openreseurope.15400.r28319

**Published:** 2022-02-07

**Authors:** Eleonora Bargiacchi

**Affiliations:** 1 IMDEA Energy Institute, Madrid, Spain

The paper investigates the integration of Power to Gas (PtG) technology into oxygen blast furnaces (OBF). For this purpose, a corrected version of the Rist diagram is proposed to predict the system operating conditions. The major novelties of the paper rely on the revisitation of the blast operating diagram and on the integration of OBF and PtG technologies.

 The paper is novel, well organized, clear, and scientifically robust. I hereby suggest some modifications and corrections that the authors may wish to implement in future versions of the paper:

- In the introduction, a better contextualization of the advantages and disadvantages of OBF with respect to other technologies would be useful to the reader. Similarly, the proposed solution (OBF+PtG) should be better contextualized (advantages/disadvantages with respect to other solutions). Overall, some useful references proposing advances in steelmaking plants with respect to carbon capture and emission reduction should be added (e.g.: Arasto *et al.*)
- "Within this framework, several authors have studied the application of power to gas (PtG) to ironmaking processes based on the reduction of iron ores with coke in a blast furnace (BF)." The references to these authors/papers are missing.
- After the integration of the blast furnace with the PtG plant the author states: "Thus, in overall terms, the CO _2_ is diminished by 71 kg _CO2_/t _HM_ with respect to the base case by using 1.93 GJ/t _HM_ of electricity in the electrolyzer, so the energy penalty of the CO _2_ avoidance is 27.1 MJ/kg _CO2_. ". Are the impacts associated to the electricity supplied to the electrolyzer accounted for? If not, I recommend including a discussion on the impacts of the electricity supplied to the electrolysis process. Also, the avoided CO2 emissions and CO2 emissions avoidance ratio should be recalculated by adding the impacts of the electrolysis (and table 5 should be modified accordingly).

Minor comment:

- I recommend the use of the simple past instead of the present perfect (e.g.: in the "Conclusions" section).

Is the study design appropriate and does the work have academic merit?

Yes

Is the work clearly and accurately presented and does it cite the current literature?

Yes

If applicable, is the statistical analysis and its interpretation appropriate?

Not applicable

Are all the source data underlying the results available to ensure full reproducibility?

Yes

Are the conclusions drawn adequately supported by the results?

Yes

Are sufficient details of methods and analysis provided to allow replication by others?

Yes

Reviewer Expertise:

Power-to-Fuel and hydrogen technologies

I confirm that I have read this submission and believe that I have an appropriate level of expertise to confirm that it is of an acceptable scientific standard.

## References

[1] : Oxygen blast furnace with CO 2 capture and storage at an integrated steel mill—Part I: Technical concept analysis. *International Journal of Greenhouse Gas Control* .2014;30: 10.1016/j.ijggc.2014.09.004 140-147 10.1016/j.ijggc.2014.09.004

# Peer review: Reviewer response for version 1

**DOI:** 10.21956/openreseurope.15400.r28384

**Published:** 2022-02-01

**Authors:** Johannes Schenk

**Affiliations:** 1 Montanuniversität Leoben, Leoben, Austria

Generations of blast furnace operators use the Rist diagram to evaluate the operating condition of their furnace. With this publication, the authors have shown that this diagram can also be used to evaluate new alternative process variants for the blast furnace. They have extended the original model approach of Rist to make the application more universal. In particular, the following extensions were made: (1) nearly any auxiliary injection composition and material is possible, (2) a new model approach for the energy balance that takes into account all possible energy sinks and sources, (3) for the operating line, the chemical efficiency is taken into account for the position relative to the Wüstit point W and in the determination of the energy balance point P.

 With this publication, the authors have made a significant contribution to the future application of the Rist diagram for the blast furnace process. The published model approach is universal. A valuable contribution is the detailed description of submodels in appendixes A to D. In the case studies presented, new blast furnace process and integration concepts are investigated and evaluated. These results are scientifically and technologically new. I appreciate reviewing the paper.

 My recommendation is to approve this publication as fit to pass peer review.

 I have some comments and questions that the authors might consider in the final version:

- Equation (49) - The variable т _j _is not clear. Is the “+” operator after т_j correct, or should it be “x”?
- Final degree of oxidation of BFG, x _i - _Is the LOI and moisture of burden and coke considered?
- Calculation of flame temperature: Which temperature is assumed for the coke entering the raceway?

Is the study design appropriate and does the work have academic merit?

Yes

Is the work clearly and accurately presented and does it cite the current literature?

Yes

If applicable, is the statistical analysis and its interpretation appropriate?

Not applicable

Are all the source data underlying the results available to ensure full reproducibility?

Yes

Are the conclusions drawn adequately supported by the results?

Yes

Are sufficient details of methods and analysis provided to allow replication by others?

Yes

Reviewer Expertise:

My area of expertise is ironmaking. I am dealing with blast furnace, direct reduction and smelting reduction technologies.

I confirm that I have read this submission and believe that I have an appropriate level of expertise to confirm that it is of an acceptable scientific standard.
